# High mitochondrial DNA content is a key determinant of stemness, proliferation, cell migration, and cancer metastasis in vivo

**DOI:** 10.1038/s41419-024-07103-9

**Published:** 2024-10-11

**Authors:** Marta Mauro-Lizcano, Filippo Di Pisa, Luis Larrea Murillo, Conor J. Sugden, Federica Sotgia, Michael P. Lisanti

**Affiliations:** 1 Translational Medicine, School of Science, Engineering and Environment (SEE), University of Salford, Greater Manchester, M5 4WT UK; 2 Present Address: Lunella Biotech, 1145 Carling Avenue, Ottawa, ON K1Z 7K4 Canada

**Keywords:** Cancer stem cells, Cancer metabolism

## Abstract

Here, we examined the potential role of mitochondrial DNA (mtDNA) levels in conveying aggressive phenotypes in cancer cells, using two widely-used breast cell lines as model systems (MCF7[ER+] and MDA-MB-231[ER-]). These human breast cancer cell lines were fractionated into mtDNA-high and mtDNA-low cell sub-populations by flow cytometry, using SYBR Gold as a vital probe to stain mitochondrial nucleoids in living cells. Enrichment of mtDNA-high and mtDNA-low cell sub-populations was independently validated, using a specific DNA-binding mAb probe (AC-30-10), and mitochondrial-based functional assays. As predicted, mtDNA-high MCF7 cells showed significant increases in mitochondrial mass, membrane potential, and superoxide production, as well as increased mitochondrial respiration and ATP production. Moreover, mtDNA-high MCF7 cells demonstrated increases in stemness features, such as anchorage-independent growth and CD44 levels, as well as drug-resistance to Gemcitabine and Tamoxifen. Proliferation rates were also significantly increased, with a dramatic shift towards the S- and G2/M-phases of the cell cycle; this was indeed confirmed by RNA-Seq analysis. Complementary results were obtained with MDA-MB-231 cells. More specifically, mtDNA-high MDA-MB-231 cells showed increases in stemness features and ATP production, as well as rapid cell cycle progression. Moreover, mtDNA-high MDA-MB-231 cells also exhibited increases in both cell migration and invasion, suggesting a role for mtDNA in distant metastasis. To test this hypothesis more directly, a preclinical in vivo model was utilized. For this purpose, MDA-MB-231 tumour cell grafts were treated with an established mtDNA synthesis inhibitor, namely Alovudine (3’-deoxy-3’-fluorothymidine). As expected, drug-induced depletion of mtDNA led to a shift from mitochondrial to glycolytic metabolism. Interestingly, Alovudine very effectively reduced the formation of spontaneous metastases by nearly 70%, but minimally inhibited tumour growth by approximately 20%. Taken together, these data suggest that high mtDNA content is a key driver of stemness, proliferation, and migration, as well as cancer cell metastasis.

## Introduction

Mitochondria are essential organelles, which play a key role in a variety of metabolic processes, and especially in oxidative phosphorylation (OXPHOS), to generate ATP [1–5]. Unlike most other membranous organelles, mitochondria have a double-membrane. The outer membrane separates the mitochondrion from the surrounding cytosol. The inner membrane invaginates inwardly, thereby forming lamellar cristae, that are covered by the five supra-molecular complexes involved in cellular respiration. Mitochondrial organelles also contain their own genome, namely mitochondrial DNA (mtDNA) [1–5].

mtDNA exists as a double-stranded, circular molecule of ~16.6 kb, which encodes 37 genes: two rRNAs (12S and 16S), 22 tRNAs, and 13 proteins. These 13 polypeptides are all subunits of the oxidative phosphorylation machinery (namely, Complexes I-V). Mutation, deletion, or depletion of mtDNA can cause a spectrum of mitochondrial diseases and have been implicated in aging and cancer [3–7]. mtDNA is located in the mitochondrial matrix, and there are multiple identical copies within each mitochondrion [8]. Both i) mtDNA copy number and ii) the specific sequence of the mtDNA, are important determinants of cell behaviour and human mitochondrial disease phenotypes [9].

It has been described that mtDNA molecules are compacted into nucleo-protein complexes, known as *“mitochondrial nucleoids”*. Most mammalian mitochondrial nucleoids contain just a single copy of mtDNA. Moreover, an increase in mtDNA copy number results in an increase in mitochondrial nucleoid number, but not their size. Many aspects of the regulation and distribution of mitochondrial nucleoids still remain unknown, as well as how mtDNA copy number is controlled [10, 11].

mtDNA alterations have been reported in a variety of cancers [6, 7]. However, the role of the mtDNA content in cancer cells, and specifically in cancer stem cells (CSCs), has not been widely studied. CSCs are a sub-population of cells with stem cell-like features that are considered to be responsible for tumour heterogeneity, cancer recurrence, treatment failure, and metastatic dissemination. CSCs also show other aggressive characteristics, such as 3D anchorage-independent growth, self-renewal, tumour initiation, high proliferation rates, and/or drug-resistance. Accordingly, the elimination of CSCs represents one of the most important new therapeutic approaches in cancer treatment [12]. In this context, mtDNA content could represent a novel mitochondrial target for intervention [7, 13–16].

Currently, several different techniques can be used to label, visualize, and/or quantitatively describe the characteristics of mtDNA. Among them are microscopy techniques, DNA-binding dyes, nucleotide analogues, antibodies and/or in situ hybridization. Focusing on the DNA-binding dyes, a wide variety of them exist that can label DNA, used mostly to detect nuclear DNA, but only a few of them have been reported to preferentially label mtDNA, under specific conditions [17]. For example, SYBR Gold is a well-established and highly sensitive nucleic acid stain. Jevtic et al. described that SYBR Gold accumulates only in living cells and specifically in the mitochondria, with an intact membrane potential [18]. They observed that low concentrations of SYBR Gold preferentially stain mitochondrial nucleoids (at a dilution of 1:10,000), while higher concentrations stain both genomic DNA and mitochondrial DNA [18]. As such, SYBR Gold can be used for vital imaging of mitochondrial DNA-containing nucleoids in living cells.

Here, we took advantage of this property and extended the use of SYBR Gold, as a new probe for flow cytometry, to isolate different cell sub-populations, with higher- or lower- amounts of mtDNA. This allowed us to evaluate the possible functional relationship between mtDNA content and stemness, as well as other aggressive properties, in cancer cells. Complementary experiments were also performed with, Alovudine, an established inhibitor of mtDNA synthesis, which targets POLG1 [19].

## Materials and methods

### Cell models and other reagents

MCF7 and MDA-MB-231 cells were purchased from the American Type Culture Collection (ATCC). Cells were cultured in Dulbecco’s Modified Eagle Medium (DMEM; Sigma-Aldrich, Darmstadt, Germany, #D6546), supplemented with 10% Fetal Bovine Serum (HI-FBS; Gibco, Massachusetts, USA, #10082-147), 2 mM Glutamax (Gibco, #35050-061), and 1% Penicillin-Streptomycin (Sigma-Aldrich, #P0781). Cells were grown at 37 °C in a 5% CO_2_ humidified incubator. Gemcitabine and 4-OH-Tamoxifen were obtained from Sigma-Aldrich (#579002, #G6423). Alovudine was obtained from ChemScene (New Jersey, USA, #CS-0013305).

### SYBR gold staining and cell isolation

Mitochondrial nucleoids in MCF7 and MDA-MB-231 cells were stained with SYBR Gold dye (ThermoFisher, Massachusetts, USA, #S11494) at a dilution of 1:20,000 for 30 min. After staining, the cells were subjected to flow cytometry, using the SONY SH800 Cell Sorter, to isolate the 5% highest green fluorescent (mtDNA-high 5%) and the 5% lowest green fluorescent (mtDNA-low 5%) sub-populations (Supplementary Fig. S1). These sub-populations were then subjected to different functional assays, to characterize their phenotypic differences experimentally.

### RT-qPCR analysis

RNA was extracted using Monarch Total RNA Miniprep Kit (New England Biolabs, Massachusetts, USA, #T2010S) following the manufacturer’s instructions for cultured mammalian cells, and samples were diluted to a final concentration of 20 ng/μl.

Luna Universal One-Step RT-qPCR Kit (New England Biolabs, #E3005S) was used for RT-qPCR reactions. Briefly, a 10 μL reaction contained: 5 μL Reaction MasterMix, 0.5 μL EnzymeMix, 0.4 μL primers (10 μM, Fwd + Rv mixed), 1 μL of RNA (20 ng), and 3.1 μL water. RT-qPCR reactions were performed in triplicate, and three biological repeats were analysed for each condition. RT-qPCRs were performed in a StepOnePlus™ Real-Time PCR System (Applied Biosystems, Massachusetts, USA) with conditions as follows: 55 °C for 10 min, 95 °C for 1 min, followed by 45 cycles of 95 °C for 10 s, 60 °C for 30 s. The cycle threshold value (Ct) was calculated for each sample and normalized separately to GAPDH and UBC. The relative expression levels were calculated using ΔΔCT method using the geometric mean of the reference transcripts [20]. Primer sequences and melt curves can be found in Supplementary Fig. S2.

### AC-30-10 immuno-staining

Fifty thousand cells were plated in 96-well-plates and incubated in a humidified atmosphere at 37 °C and 5% CO_2_ for 24 h after sorting. After incubation, cells were fixed with 4% PFA for 15 min at room temperature (RT), permeabilizated with Triton 0.2% for 15 min at room temperature and blocked with 3% BSA-PBS for 45 min at RT. Subsequently, cells were incubated with the primary antibody AC-30-10 (Progen, Heidelberg, Germany, #690014) at a dilution of 1:200 for 1 h at room temperature, followed by incubation with the secondary antibody Goat Anti-Mouse IgM mu chain (Alexa Fluor® 647) (Abcam, Cambridge, UK, #ab150123) at a dilution of 1:500 for 1 h at room temperature. Nuclei were counter-stained with Hoechst 33342 trihydrochloride (Sigma-Aldrich, #H3570). Images were acquired with an EVOS automated microscope and analysed and quantified with ImageJ using the macro and protocol specified in Supplementary Fig. S3A–C.

### MT-CO1 and MT-CO2 immuno-staining

After sorting with SYBR Gold, 50,000 cells were plated onto a glass slide (12-well chamber removable; Thistle Scientific, Glasgow, UK, #81201) and incubated in a humidified atmosphere at 37 °C and 5% CO_2_ for 24 h after sorting. Cells were fixed with 4% PFA for 15 min at RT, permeabilizated with Triton 0.2% for 15 min at RT and blocked with 3% BSA- PBS for 45 mins at RT. Subsequently, the cells were incubated with 1:500 dilution of Alexa Fluor 647 Anti-MTCO1 antibody (1D6E1A8) (Abcam, Cambridge, UK, #ab198600) and a 1:50 dilution of Alexa Fluor 647 Anti-MTCO2 antibody (EPR3314) (Abcam, #ab200525) for 1 h at RT. Cell nuclei were stained with DAPI (Vectashield Antifade Mounting Medium with DAPI; Vector Laboratories, California, USA, #H-1200). Images were acquired with EVOS automated microscope and analysed and quantified with ImageJ using the macro and protocol specified in Supplementary Fig. S3A–C.

### MT-CO2 analysis

After sorting with SYBR Gold, cells were fixed with 2% PFA, permeabilizated with 0.1% Triton and incubated with a 1:200 dilution of Alexa Fluor 647 Anti-MT-CO2 antibody (EPR3314) (Abcam, #ab200525) for 1 h at RT. Cells were washed and resuspended in PBS, and analysed by FACS (Attune ^TM^ NxT Flow Cytometer, ThermoFisher Scientific).

### ATP Assay using Cell-Titer-Glo 2.0

Cell Titer Glo 2.0 was obtained from Promega (Wisconsin, USA, #G9242). Briefly, 10,000 cells were seeded after sorting into a 96-well plate and cultured in complete DMEM medium and incubated with the Cell-Titer-Glo 2.0 Reagent in a humidified atmosphere at 37 °C and 5% CO_2_ for 15 min. Luminescence content was evaluated using the Varioskan ^TM^ LUX plate reader (ThermoFisher Scientific).

### Mammosphere formation assay

A single cell suspension was prepared after sorting using enzymatic (1x Trypsin-EDTA; Sigma Aldrich, #T3924), and manual disaggregation (25-gauge needle). Then, 5000 cells per well were plated into mammosphere medium (DMEM-F12, Gibco, #21041-025; B27, Gibco, #17504-044; 20 ng/ml EGF, Peprotech, Massachusetts, USA, #AF-100-15; 1% PenStrep) under non-adherent conditions, in 6-wells-plates coated with Poly 2-hydroxyethyl methacrylate (poly-HEMA, Sigma-Aldrich, #P3932). Alovudine was incubated at the indicated concentrations in the mammosphere medium during the 5 days. Mammospheres greater than 50μm were counted, using an eye-piece graticule, after 5 days of incubation.

### CD44 analysis

After sorting with SYBR Gold, cells were incubated with a CD44 antibody (CD44 Monoclonal Antibody (IM7), APC, Thermo-Fisher Scientific, #17-0441-83) or the isotype antibody control (Rat IgG2b kappa Isotype Control, APC, Thermo-Fisher Scientific, #17-4031-82) for 30 min on ice after sorting. Cells were washed and resuspended in 1% BSA-PBS, and analysed by flow cytometry (Attune^TM^ NxT Flow Cytometer, ThermoFisher Scientific).

### xCELLigence RTCA system

After sorting with SYBR Gold, cell growth was evaluated using the xCELLigence Real-Time Cell Analysis (RTCA) system (Agilent, California, USA), via the measurement of cell-induced electrical impedance. Briefly, 10,000 cells per well were seeded into RTCA E-Plates (Agilent, #300601140), and cell growth (Slope) was followed for approximately 40 hours.

### Cell cycle analysis

Analysis of the cell cycle was carried out using the Muse® Cell Cycle Kit (Luminex, Massachusetts, USA, #MCH100106). After sorting, cells were incubated with propidium iodide for 20 min and analysed by flow cytometry (Attune ^TM^ NxT Flow Cytometer, ThermoFisher Scientific).

### Seahorse XFe-96 metabolic flux analysis

To evaluate real-time Oxygen Consumption Rates (OCRs) and Extracellular Acidification Rates (ECARs), we used the Seahorse Extracellular Flux (XFe96) analyser (Agilent/Seahorse Bioscience). After sorting, 50,000 cells per well were seeded in the XFe-96-well plates and incubated in a humidified atmosphere at 37 °C and 5% CO_2_ for 24 h. In the Alovudine seahorse assays, after 6-days of pre-treatment with Alovudine at the indicated concentrations, 15,000 cells were seeded in the XFe-96-well plates and incubated in a humidified atmosphere at 37 °C and 5% CO_2_ for 24 h. Cells were incubated in 175 μL/well of XF assay media at 37 °C, in a non-CO_2_ incubator for 1 h before the Seahorse assay. The XFe-96 sensor cartridge was loaded with 25 μL of 80 mM glucose, 9 μM oligomycin and 1 M 2-deoxyglucose (for ECAR measurements) or 10 μM oligomycin, 10 μM FCCP, 10 μM rotenone and 10 μM antimycin A (for OCR measurements) in XF assay media. Measurements were normalized by Hoechst staining. Data sets were analysed using XFe-96 software.

### Mitochondrial analysis with fluorescent probes

After sorting with SYBR Gold, cells were stained with MitoTracker Deep Red (Invitrogen, #M22426), or mtSOX Deep Red (Dojindo, Japan, #MT16-12) for 30 min at 37 °C. Alternatively, cells were stained with MitoNIR Dye (NIR, Mitochondrial Membrane Potential Assay Kit; Abcam, #ab112149) for 30 min at RT. After staining, cells were washed and resuspended in PBS, and analysed by flow cytometry (Attune^TM^ NxT Flow Cytometer, ThermoFisher Scientific).

### Cell migration and invasion assays

For both cell migration and invasion experiments, 24-well Transwell tissue culture inserts, with an 8μm pore size, PET membrane, were utilized. However, uncoated transparent membranes were used for cell migration (Corning, New York, USA, #353097), while extracellular matrix (Matrigel) pre-coated membranes were used for cell invasion (Corning, #354480). In both types of experiments, 50,000 cells were plated after sorting into the upper chamber of the Transwell in a serum-free DMEM with 1% Penicillin-Streptomycin. In the case of the Alovudine assays, 30,000 cells were plated after Alovudine 6-days pretreatment into the upper chamber of the Transwell in a serum-free DMEM with 1% Penicillin-Streptomycin. The lower chamber was filled with complete culture medium (DMEM with 10% FBS, 2 mM Glutamax, and 1% Penicillin-Streptomycin) as the chemo-attractant. Cells were incubated in a humidified atmosphere at 37 °C and 5% CO_2_ for 6 h in the migration experiments and 16 h in the invasion experiments. Cells were removed from the upper surface by scrubbing with cotton swabs. Chambers were fixed with ethanol 70% and stained in 0.5% crystal violet (Sigma-Aldrich, # HT90132), for 15 min each, rinsed in water, and examined under a bright-field microscope. Values were obtained by counting the whole membrane in the sorting experiments, given the low number of migrating cells. By contrast, in the Alovudine experiments it was necessary to obtain the values by counting five fields per membrane (20X objective), given the high number of migrating cells.

### Quantitating the relative abundance of mtDNA

DNA extractions were performed using the Monarch® Genomic DNA Purification Kit (New England Biolabs, #T3010S) from cell pellets of 1 ×10^6^ cells. DNA was subsequently diluted to a final concentration of 5 ng/μl. Relative mitochondrial DNA copy number was obtained by using the Relative Human Mitochondrial DNA Copy Number Quantification qPCR Assay Kit (ScienCell, California, USA, #8938). Briefly, a 10 μL reaction contained: 5 μL Reaction MasterMix, 1 μL primers (10 μM, Fwd + Rv mixed), 1 μL of DNA (5 ng) and 3 μL water. qPCR reactions were performed in duplicate or triplicate, and three biological repeats were analysed for each condition. qPCRs were performed in a StepOnePlus™ Real-Time PCR System (Applied Biosystems), with conditions as follows: 95 °C for 10 min, followed by 32 cycles of 95 °C for 20 s, 52 °C for 20 s, and 72 °C for 45 s. The relative mtDNA levels were calculated using the ΔΔCT method [20].

### Evaluating the in vivo efficacy of alovudine: tumour growth, metastasis, and toxicity

To evaluate in vivo efficacy of Alovudine in preventing tumour growth and metastasis, we employed MDA-MB-231 cells in an in vivo pre-clinical model, known as the CAM (chorio-allantoic membrane) assay. Briefly, fertilized eggs are incubated at 37.5 °C, with 50% relative humidity, for 9 days. On embryonic day 9 (E9), the CAM is dropped down by drilling a small hole through the eggshell into the air sac, and at least 15 eggs are grafted with MDA-MB-231 cells for each group. MDA-MB-231 cells were first cultured and expanded in DMEM with 10% FBS and 1% Penicillin-Streptomycin. Next, MDA-MB-231 cells were detached with trypsin and resuspended in graft medium. One million of cells were added onto the CAM of each egg. On day E10, tumours were detectable, and they were then treated daily for 9 days with vehicle alone (0.5% DMSO in PBS) or with three different dosages of Alovudine (50, 100 and 250 μM). On day E18, an evaluation of tumour growth, metastatic invasion, and embryonic toxicity were assessed (Supplementary Fig. S4). Alovudine treatment was compared with the vehicle alone control.

The CAM assay was performed by INOVOTION (Société: 811310127), La Tronche-France. According to the French legislation, no ethical approval is needed for scientific experimentations using oviparous embryos (decree n° 2013-118, February 1, 2013; art. R-214-88). Animal studies were performed under animal experimentation permits N° 381029 and B3851610001 to Jean Viallet (INOVOTION).

### Statistical analysis

All analyses were performed with GraphPad Prism 10 software. Statistical significance was determined using the Student’s *t* test or ANOVA test. Data are shown as the mean ± SD (or ±SEM for in vivo experiments). All in vitro experiments were performed at least three times independently, with three or more technical replicates for each experimental condition tested, as specified in the legend of each figure, unless stated otherwise. Values of *P* < 0.05 were considered significant, where *P < 0.05, **P < 0.01, ***P < 0.001, ****P < 0.0001, ns not significant (*P* > 0.05).

## Results

### Enrichment of mtDNA-high and mtDNA-low cell sub-populations by flow cytometry, using SYBR Gold vital staining of mitochondrial DNA-containing nucleoids

Here, we investigated the potential relationship between mitochondrial DNA (mtDNA) content and aggressive cell phenotypes, using two breast cancer cell lines as a model system (MCF7[ER+] and MDA-MB-231[ER-]). For this purpose, we used SYBR Gold, a nucleic acid dye, to obtain two cell sub-populations, based on its green fluorescence intensity, using flow cytometry (Supplementary Fig. S1). Low concentrations of SYBR Gold preferentially and specifically recognize mitochondrial DNA nucleoids, but not nuclear DNA. SYBR Gold also shows a large fluorescence enhancement upon binding to nucleic acids (approximately 1000-fold). As such, cells with higher levels of SYBR Gold fluorescence intensity are predicted to have higher levels of mtDNA, relative to cells with lower levels of fluorescence intensity.

**Fig. 1:**
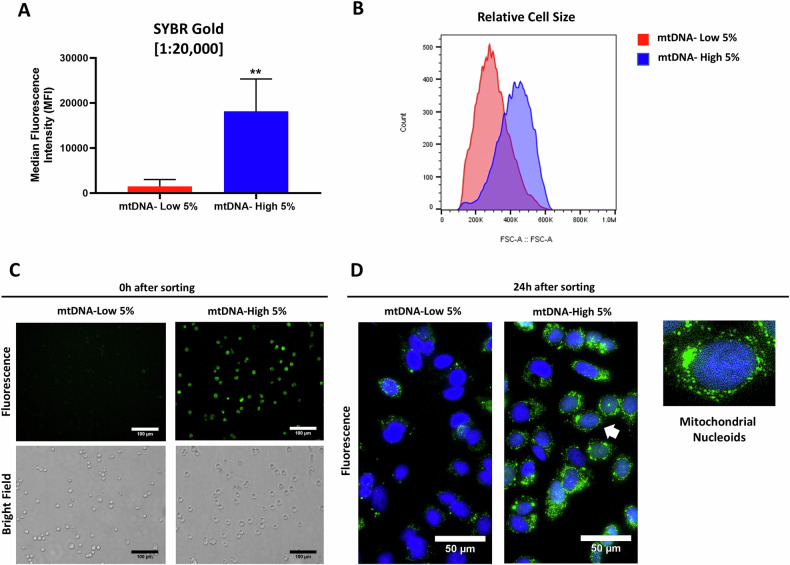
SYBR Gold dye permits the enrichment of mtDNA-high and mtDNA-low cell sub-populations by flow cytometry, based on green fluorescence intensity levels in MCF7 cells. MCF7 cells were incubated for 30 min with low concentrations of SYBR Gold dye (at a dilution of 1:20,000). After cell staining, samples were subjected to FACS sorting to isolate the 5% highest (mtDNA-high 5%) and the 5% lowest (mtDNA-low 5%) green fluorescent cell sub-populations. **A** Median fluorescence intensity (MFI) of the mtDNA-high 5% and mtDNA-low 5% sub-populations by FACS analysis. Data are shown as the mean ± standard deviation (SD) (*n* = 4). Statistical significance was determined using an unpaired Student’s *t* test, **p ≤ 0.01. **B** Relative cell size of the mtDNA-high 5% cells and mtDNA-low 5% cells by FACS. A representative experiment is shown. Fluorescent microscopy of the SYBR Gold stained cell sub-populations (mtDNA-high 5% cells vs. mtDNA-low 5%), either (**C**) immediately after sorting (0 h) or (**D**) 1 day after sorting (24 h). Note the appearance of fluorescent green-stained mitochondrial nucleoids, containing mtDNA. An arrowhead points at a single cell, which is also shown as an *Inset*.

More specifically, we stained MCF7 cells with SYBR Gold for 30 min at a dilution of 1:20,000, as previous studies reported that low concentrations of SYBR Gold only stained mitochondrial DNA [18]. As shown in Fig. 1A, we obtained two cell sub-populations: i) one with high levels of mtDNA (mtDNA-high 5%) and ii) another with low levels of mtDNA (mtDNA-low 5%), based on a > 20-fold difference in SYBR Gold fluorescence intensity. Furthermore, these two MCF7 sub-populations exhibited different cell sizes (Fig. 1B). Also, note that SYBR Gold staining is non-toxic for the cells (Supplementary Fig. S5).

We validated that we obtained two distinctly-stained sub-populations by fluorescence microscopy, immediately following cell sorting (Fig. 1C). Furthermore, 24 hours after plating, the attached mtDNA-high cells showed an abundance of mitochondrial nucleoids (Fig. 1D, Right). In contrast, the mtDNA-low cells showed a paucity, or even an absence, of mitochondrial nucleoids (Fig. 1D, Left).

**Fig. 2:**
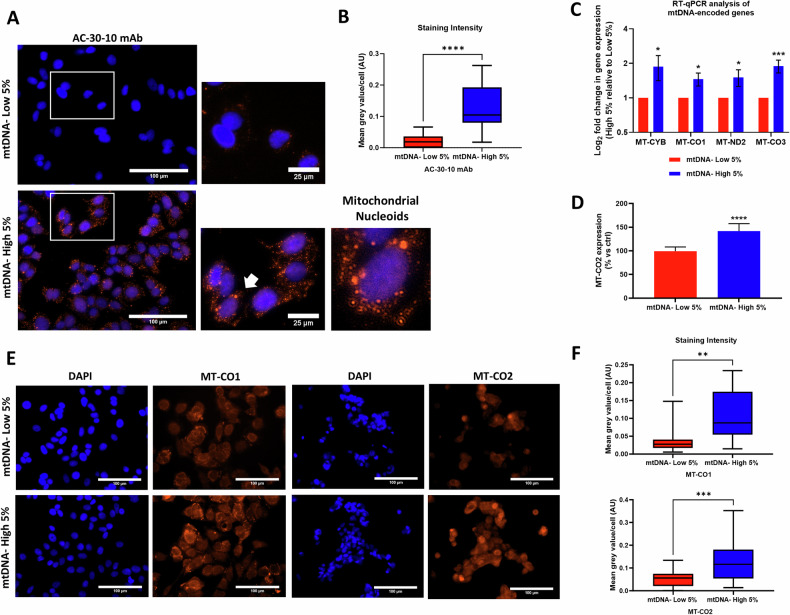
mtDNA-high MCF7 cells show higher levels of mitochondrial DNA, mRNAs and proteins. MCF7 cells were incubated for 30 min with low concentrations of SYBR Gold dye (at a dilution of 1:20,000). After cell staining, samples were subjected to FACS sorting to isolate the 5% highest (mtDNA-high 5%) and the 5% lowest (mtDNA-low 5%) green fluorescent cell sub-populations. **A** Cells were immuno-stained with a mouse monoclonal antibody (AC-30-10 mAb) that preferentially recognizes mtDNA. Note the appearance of fluorescent red-stained mitochondrial nucleoids, containing mtDNA. **B** Mean grey intensity values of AC-30-10 mAb staining. Each box and whisker plot represents the mean ± SD of the quantification of the mean staining intensity per cell, derived from 12 fields of view (mtDNA-low 5%) or 19 fields of view (mtDNA-high 5%). Quantitation of the images revealed a 6.2-fold increase in the differential fluorescence intensity, in mtDNA-high cells relative to mtDNA-low cells. Each field of view comprises an average of 66 cells, and the data were collected from three distinct batches of staining. Statistical significance was determined using an unpaired Student’s *t* test, ****p ≤ 0.0001. **C** Reverse transcription quantitative polymerase chain reaction (RT-qPCR) analyses of mtDNA-high 5% cells compared to mtDNA-low 5% cells. Values were normalized to GAPDH and UBC and graphed as log fold change relative to geomean of the two reference transcripts of the mtDNA-low 5% condition. Bars represent the mean ± SD (*n* = 3). Statistical significance was determined using an unpaired Student’s *t* test, * *p* ≤ 0.05, ***p ≤ 0.001. **D** MT-CO2 protein levels were assessed by FACS. Data are shown as mean ± SD (*n* = 4) representing the mtDNA-high 5% increase over mtDNA-low 5% cells. Statistical significance was determined using an unpaired Student’s *t* test, ** *p* ≤ 0.01. **E** Immuno-staining with antibodies directed against MT-CO1 and MT-CO2 proteins. **F** Mean grey intensity values of MT-CO1 and MT-CO2 immuno-staining. Each box and whisker plot represents the mean ± SD of the quantification of the mean staining intensity per cell, derived from 8 fields of view (mtDNA-low 5%) or 12 fields of view (mtDNA-high 5%) for MT-CO1 and derived from 20 fields of view (mtDNA-low 5%) or 23 fields of view (mtDNA-high 5%) for MT-CO2. Each field of view comprises an average of 103 cells for MT-CO1 and an average of 72 cells for MT-CO2, and the data were collected from three distinct batches of staining. Quantitation of the fluorescent images revealed a 2.8-fold increase in MT-CO1 and a 2.3-fold increase in MT-CO2, in mtDNA-high cells. Statistical significance was determined using an unpaired Student’s *t* test, ** *p* ≤ 0.01, ***p ≤ 0.001.

To further stringently validate the model, we used a specific DNA-binding monoclonal antibody probe (AC-30-10 mAb), previously employed in >20 studies to stain and specifically visualize mtDNA [21, 22]. As expected, mtDNA-high cells showed higher levels of fluorescent immuno-staining for mtDNA (Fig. 2A). Furthermore, quantitation of the images revealed a 6.2-fold increase in the differential fluorescence intensity, in mtDNA-high cells relative to mtDNA-low cells (Fig. 2B). In fact, in the images, we could appreciate the appearance of typical mitochondrial DNA-containing nucleoids, as we observed with SYBR Gold staining.

We verified the mRNA expression levels of several distinct mtDNA-encoded genes, such as MT-CYB, MT-CO1, MT-ND2 and MT-CO3 by RT-qPCR, directly demonstrating that mtDNA-high cells have higher amounts of mtDNA-dependent gene expression (Fig. 2C; See also Supplementary Fig. S2). In addition, we determined the protein expression levels of MT-CO2, another mtDNA-dependent gene, by FACS analysis (Fig. 2D). Finally, both MT-CO1 and MT-CO2 exhibited higher levels of protein expression in mtDNA-high cells, as compared with mtDNA-low cells, by fluorescence microscopy (Fig. 2E). Quantitation of the fluorescent images revealed a 2.8-fold increase in MT-CO1 and a 2.3-fold increase in MT-CO2, in mtDNA-high cells (Fig. 2F). Merged images are also shown in Supplementary Fig. S3.

Taken together, these results clearly show that mtDNA-high cells also have higher levels of i) mtDNA-specific RNA and ii) mtDNA-specific protein expression, as predicted.

### mtDNA-high MCF7 cells show increases in stemness features, such as 3D-anchorage-independent growth, CSC markers, cell proliferation, and mitochondrial metabolism

Next, after having validated the system, we examined the relationship between mtDNA content and stemness features, using SYBR Gold to stain the mitochondrial nucleoids in MCF7 cells, followed by flow cytometry.

**Fig. 3:**
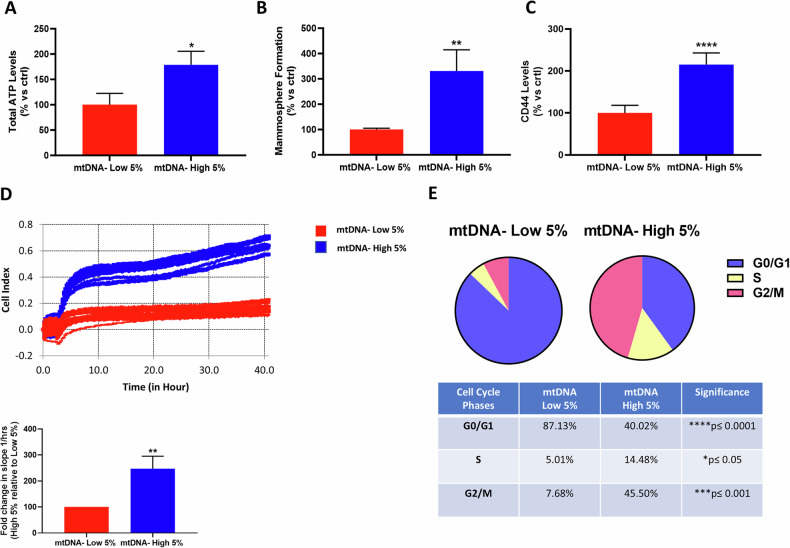
mtDNA-high MCF7 cells form mammospheres more efficiently, and show higher levels of total ATP and the stemness marker CD44, as well as increased proliferation rates. MCF7 cells were incubated for 30 min with low concentrations of SYBR Gold dye (at a dilution of 1:20,000). After cell staining, samples were subjected to FACS sorting to isolate the 5% highest (mtDNA-high 5%) and the 5% lowest (mtDNA-low 5%) green fluorescent cell sub-populations. **A** ATP levels were determined using the Cell-Titer-Glo 2.0 Reagent for 15 min in mtDNA-high 5% and mtDNA-low 5% subpopulations, plated in complete DMEM medium. Three experimental repeats, each with three technical replicates, were performed for each condition. Data are shown as the mean ± SD (*n* = 3), representing the mtDNA-high 5% increase over mtDNA-low 5% cells. Statistical significance was determined using an unpaired Student’s *t* test, * *p* ≤ 0.05. **B** Mammosphere assay. Single cells were seeded in low attachment plates and analysed after 5 days. Three experimental repeats, each with three technical replicates, were performed for each condition. Data are shown as the mean ± SD (*n* = 3), representing the mtDNA-high 5% increase over mtDNA-low 5% cells. Statistical significance was determined using an unpaired Student’s *t* test, ** *p* ≤ 0.01. **C** CD44 levels were determined with an APC mouse anti-Human CD44 by flow cytometry. Data are shown as mean ± SD (*n* = 3) representing the mtDNA-high 5% increase over mtDNA-low 5% cells. Statistical significance was determined using an unpaired Student’s *t* test, **** *p* ≤ 0.0001. **D** Proliferation was assessed using the xCELLigence RTCA system for real-time growth analysis. In the upper panel, a representative tracing is shown. In the lower panel, the slope analysis shows the average of the final cell index. Three experimental repeats, each with eight technical replicates, were performed for each condition. Data are shown as the fold change of the mtDNA-high 5% increase over mtDNA-low 5% cells. Bars represent the mean ± SD (*n* = 3). Statistical significance was determined using an unpaired Student’s *t* test, ** *p* ≤ 0.01. **E** Cell cycle was evaluated with propidium iodide by flow cytometry. The percentage of cells in G0/G1, S, and G2/M phases of the cell cycle are represented in the graphs. Data are shown as the mean ± SD (*n* = 3) representing the mtDNA-high 5% increase over mtDNA-low 5% cells. Statistical significance was determined using an unpaired Student’s *t* test, **p ≤ 0.01.

As a first step, we determined total ATP levels, as we have previously reported that high ATP levels are a key metabolic marker for stemness in cancer cells [23, 24]. As shown in Fig. 3A, we observed a near 2-fold increase in ATP levels in mtDNA-high MCF7 cells, relative to their mtDNA-low counterparts. Similarly, mtDNA-high MCF7 cells showed a 3-fold increase in their 3D anchorage-independent growth, as evidenced by the mammosphere assay (Fig. 3B). In further support of these observations, mtDNA-high MCF7 cells also showed a > 2-fold increase in CD44 levels, a well-established surface CSC marker (Fig. 3C**;** see also the cytometry plots in Supplementary Fig. S6A). However, colony formation did not show any significant changes (Supplementary Fig. S7), between the mtDNA-high and low sub-populations, indicating that this was indeed a transient phenotype, as would be expected for CSCs. In this context, it is important to note that colony formation is typically scored 2 weeks after cell plating. Similar results were obtained with Biotracker ATP-Red 1, another well-established marker for CSCs [23, 24].

In addition, we assessed cell proliferation rates, using the xCELLigence to continuously monitor cell division in real-time. Figure 3D shows that mtDNA-high MCF7 cells clearly proliferated at an increased rate, approximately 2.5-fold higher. Similarly, cell cycle analysis of mtDNA-high MCF7 cells revealed a dramatic shift from the G0/G1 phase, towards the S- and G2/M-phases of the cell cycle (Fig. 3E; see also the cytometry plots in Supplementary Fig. S6B). More specifically, there was an approximate 3-fold increase in S-phase, with a near 6-fold increase in the G2/M phase, and a corresponding >2-fold reduction in the G0/G1 phase. This phenotype of rapid cell cycle progression was further validated at the molecular level, by RNA-Seq analysis, which clearly showed that cell cycle related mRNA transcripts were upregulated in the mtDNA-high MCF7 population, which was highly significant (Supplementary Fig. S8).

**Fig. 4:**
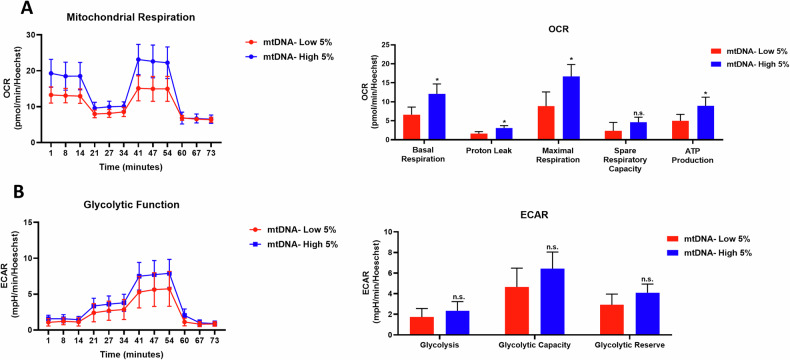
Mitochondrial respiration, but not glycolysis, is significantly increased in mtDNA-high MCF7 cells. MCF7 cells were incubated for 30 min with low concentrations of SYBR Gold dye (at a dilution of 1:20,000). After cell staining, samples were subjected to FACS sorting to isolate the 5% highest (mtDNA-high 5%) and the 5% lowest (mtDNA-low 5%) green fluorescent cell sub-populations. **A** Mitochondrial respiration was determined using the Seahorse XFe96. The left panel shows the OCR (oxygen consumption rate) tracing. In the right panel, bar graphs illustrate the levels of basal respiration, proton leak, maximal respiration, spare respiratory capacity, and ATP production, obtained from the OCR quantitation. Four experimental repeats, each of which contains four technical replicates, were performed for each condition. Data are shown as the mean ± SD (*n* = 4) Statistical significance was determined using an unpaired Student’s *t* test, *p ≤ 0.05, n.s. not significant. **B** Glycolytic function was determined using the Seahorse XFe96. The left panel shows the ECAR (extracellular acidification rate) tracing. In the right panel, bar graphs illustrate the levels of glycolysis, glycolytic capacity, and glycolytic reserve obtained from the ECAR quantification. Four experimental repeats, each of which contains four technical replicates, were performed for each condition. Data are shown as the mean ± SD (*n* = 4). Statistical significance was determined using an unpaired Student’s *t* test, n.s. not significant.

To better understand the metabolic link between mtDNA content and CSC formation, we employed metabolic flux analysis, with the Seahorse XFe96. Figure 4A shows the status of mitochondrial function, as reflected by oxygen-consumption rates (OCRs). The mitochondrial energetic profile of mtDNA-high MCF7 cells showed near 2-fold increases in basal respiration, proton leak, and maximal respiration, as well as ATP production. In contrast, mtDNA-high MCF7 cells did not show any significant changes in glycolytic function (ECAR), relative to mtDNA-low cells (Fig. 4B).

**Fig. 5:**
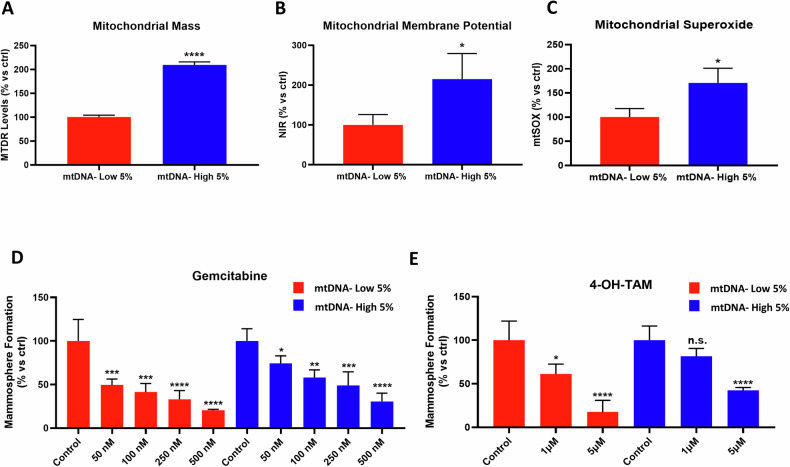
mtDNA-high MCF7 cells show significant increases in mitochondrial mass, membrane potential, and superoxide production, as well as drug-resistance. MCF7 cells were incubated for 30 min with low concentrations of SYBR Gold dye (at a dilution of 1:20,000). After cell staining, samples were subjected to FACS sorting to isolate the 5% highest (mtDNA-high 5%) and the 5% lowest (mtDNA-low 5%) green fluorescent cell sub-populations. **A** Mitochondrial mass was determined using Mitotracker Deep Red by flow cytometry. Data are shown as the mean ± SD (*n* = 3), representing the mtDNA-high 5% increase over mtDNA-low 5% cells. Statistical significance was determined using an unpaired Student’s *t* test, ****p ≤ 0.0001. **B** Mitochondrial membrane potential was evaluated using mitoNIR dye. Data are shown as the mean ± SD (*n* = 3), representing the mtDNA-high 5% increase over mtDNA-low 5% cells. Statistical significance was determined using an unpaired Student’s *t* test, *p ≤ 0.05. **C** Mitochondrial superoxide was measured using mtSOX Deep Red. Data are shown as the mean ± SD (*n* = 3), representing the mtDNA-high 5% increase over mtDNA-low 5% cells. Statistical significance was determined using an unpaired Student’s *t* test, **p ≤ 0.01. **D** mtDNA-high 5% and mtDNA-low 5% cells were seeded in low-attachment plates to evaluate mammosphere formation, with the indicated Gemcitabine concentrations. Four experimental repeats, each of which contains two technical replicates, were performed for each condition. Data are shown as the mean ± SD (*n* = 4), representing the mtDNA-high 5% increase over mtDNA-low 5% cells. Statistical significance was determined using one-way ANOVA, Dunnett’s multiple comparisons test, *p ≤ 0.05, **p ≤ 0.01, ***p < 0.001, ****p < 0.0001. **E** mtDNA-high 5% and mtDNA-low 5% cells were seeded in low-attachment plates to evaluate mammosphere formation, with the indicated 4-OH-Tamoxifen concentrations. Four experimental repeats, each of which contains two technical replicates, were performed for each condition. Data are shown as the mean ± SD (*n* = 4) representing the mtDNA-high 5% increase over mtDNA-low 5% cells. Statistical significance was determined using one-way ANOVA, Dunnett’s multiple comparisons test, *p ≤ 0.05, ****p < 0.0001, n.s. not significant.

As mitochondrial function was clearly augmented in mtDNA-high MCF7 cells, we assessed other mitochondrial-based parameters. Figure 5(panels A–C) shows that mtDNA-high MCF7 cells have an approximate 2-fold increase in mitochondrial mass, membrane potential, and superoxide production, all relative to mtDNA-low cells. Since these additional parameters are quantitatively increased, this provides further evidence that mitochondrial metabolism is clearly more active in mtDNA-high cells.

We have previously shown that increased mitochondrial function is associated with multi-drug resistance in cancer cells [25, 26]. Therefore, we examined the phenotypic sensitivity of MCF7 cells with different levels of mtDNA to two commonly used anti-cancer therapies, namely Gemcitabine (Fig. 5D) and 4-OH-Tamoxifen (Fig. 5E), using the mammosphere assay. As predicted, the mtDNA-low sub-population was clearly more sensitive to both Gemcitabine and 4-OH-Tamoxifen treatment, as compared with the mtDNA-high sub-population.

In summary, here we observed that high mtDNA content links mitochondrial energy metabolism to stemness, cell cycle progression, and drug-resistance in cancer cells.

### mtDNA-high MDA-MB-231 cells show increases in ATP, stemness features, and cell cycle progression, as well as cell migration and invasion

To further validate and extend our findings, SYBR Gold (at a dilution of 1:20,000) was used to stain mitochondrial nucleoids and to purify mtDNA-high and mtDNA-low sub-subpopulations from another commonly used breast cancer cell line, namely MDA-MB-231 cells. An advantage of using MDA-MB-231 cells is that they are a well-established model of triple negative breast cancer, that undergoes both cell migration and invasion in vitro, as well as spontaneous metastasis in vivo.

**Fig. 6:**
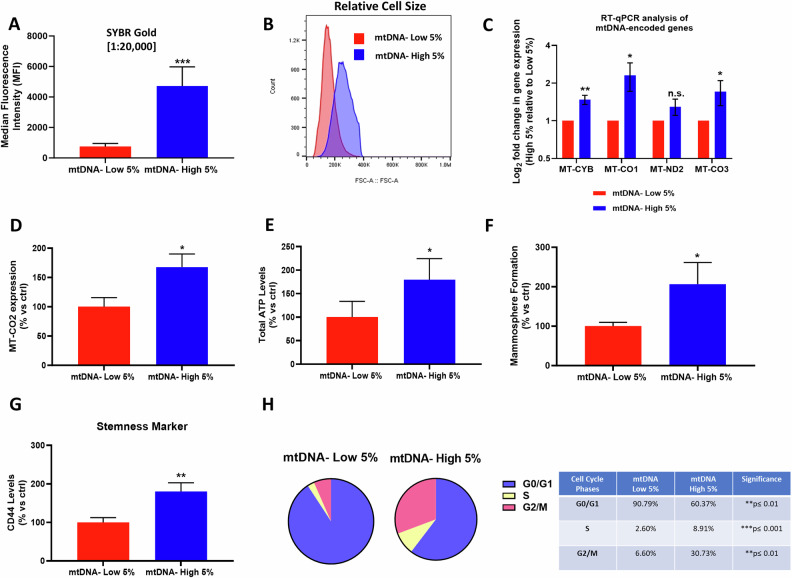
mtDNA-high MDA-MB-231 cells show increases in ATP and stemness features, as well as cell proliferation. MDA-MB-231 cells were incubated for 30 min with low concentrations of SYBR Gold dye (at a dilution of 1:20,000). After cell staining, samples were subjected to FACS sorting to isolate the 5% highest (mtDNA-high 5%) and the 5% lowest (mtDNA-low 5%) green fluorescent cell sub-populations. **A** Median fluorescence intensity (MFI) in the mtDNA-high 5% and mtDNA-low 5% subpopulations by FACS. Data are shown as the mean ± standard deviation (SD) (*n* = 4). Statistical significance was determined using an unpaired Student’s *t* test, **p ≤ 0.01. **B** Relative cell size of the mtDNA-high 5% cells and mtDNA-low 5% cells by FACS of a representative experiment. **C** Reverse transcription quantitative polymerase chain reaction (RT-qPCR) analyses of mtDNA-high 5% cells compared to mtDNA-low 5% cells. Values were normalized to GAPDH and UBC and graphed as log fold change, relative to geomean of the two reference transcripts of the mtDNA-low 5% condition. Bars represent the mean ± SD (*n* = 3). Statistical significance was determined using an unpaired Student’s *t* test, *p ≤ 0.05, **p ≤ 0.01, n.s. not significant. **D** MT-CO2 levels were assessed by FACS. Data are shown as the mean ± SD (*n* = 3), representing the mtDNA-high 5% increase over mtDNA-low 5% cells. Statistical significance was determined using an unpaired Student’s *t* test, * *p* ≤ 0.05. **E** ATP levels in mtDNA-high 5% cells and mtDNA-low 5% cells were determined using Cell Titer Glo. Four experimental repeats, each of which contains three technical replicates, were performed for each condition. Data are shown as the mean ± SD (*n* = 4) representing the mtDNA-high 5% increase over mtDNA-low 5% cells. Statistical significance was determined using an unpaired Student’s *t* test, * *p* ≤ 0.05. **F** Mammosphere formation was determined after 5 days of growth in low-attachment plates. Three experimental repeats, each of which contains three technical replicates, were performed for each condition. Data are shown as the mean ± SD (*n* = 4) representing the mtDNA-high 5% increase over mtDNA-low 5% cells. Statistical significance was determined using an unpaired Student’s *t* test, *p ≤ 0.05. **G** CD44 levels were determined with an APC mouse anti-Human CD44 antibody by flow cytometry. Data are shown as mean ± SD (*n* = 3) representing the mtDNA-high 5% increase over mtDNA-low 5% cells. Statistical significance was determined using an unpaired Student’s *t* test, *p ≤ 0.05. **H** Cell cycle was evaluated with propidium iodide by flow cytometry. The percentage of cells in G0/G1, S, and G2/M phases of the cell cycle are represented in the pie graphs. Data are shown as the mean ± SD (*n* = 3), representing the mtDNA-high 5% increase over mtDNA-low 5% cells. Statistical significance was determined using an unpaired Student’s *t* test, **p < 0.01, ***p < 0.001.

As shown in Fig. 6A, we obtained two MDA-MB-231 cell sub-populations: i) one with high levels of mtDNA (mtDNA-high 5%) and ii) another with low levels of mtDNA (mtDNA-low 5%), based on a > 10-fold difference in SYBR Gold fluorescence intensity. Furthermore, these two sub-populations exhibited different cell sizes (Fig. 6B). Moreover, mtDNA-high MDA-MB-231 cells showed increased levels of mitochondrial gene expression by RT-qPCR (Fig. 6C) or protein expression by FACS (Fig. 6D), demonstrating that this sub-population is indeed enriched in mtDNA content, as we observed earlier for MCF7 cells.

Next, we examined the relationship between mtDNA content and stemness features, using SYBR Gold to stain mitochondrial nucleoids in MDA-MB-231 cells, followed by flow cytometry. To investigate the stemness phenotype, first we determined the total ATP levels, using the Cell Titer Glo assay. Figure 6E shows that there was a near 2-fold increase in ATP levels in the mtDNA-high MDA-MB-231 sub-population.

Using the mammosphere assay, mtDNA-high MDA-MB-231 cells showed a 2-fold increase in their 3D anchorage-independent growth (Fig. 6F). Similarly, mtDNA-high MDA-MB-231 cells showed a near 2-fold increase in CD44 levels, a known plasmalemmal CSC marker (Fig. 6G; see also the cytometry plots in Supplementary Fig. S6A). However, no differences were observed in the colony formation assay, between the mtDNA-high and low sub-populations (Supplementary Fig. S7B).

Importantly, cell cycle analysis showed that mtDNA-high MDA-MB-231 cells underwent rapid cell cycle progression, with a significant decrease in the G0/G1 phase, and corresponding increases in the S- and G2/M-phases (Fig. 6H; see also the cytometry plot in Supplementary Fig. S6B).

**Fig. 7:**
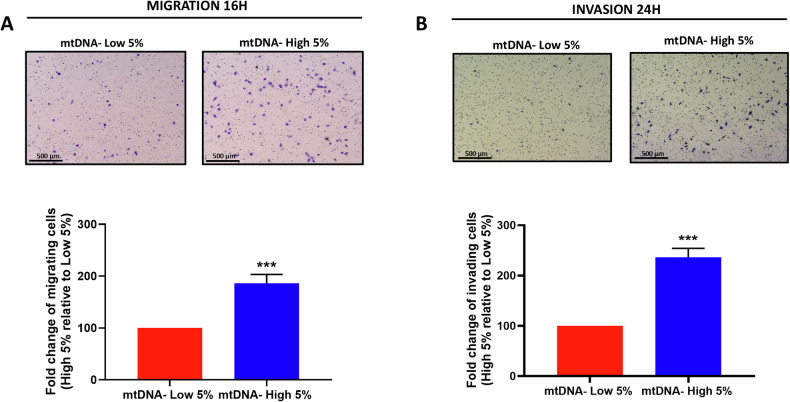
Cell migration and invasion capacity are increased in mtDNA-high MDA-MB-231 cells. MDA-MB-231 cells were incubated for 30 min with low concentrations of SYBR Gold dye (at a dilution of 1:20,000). After cell staining, samples were subjected to FACS sorting to isolate the 5% highest (mtDNA-high 5%) and the 5% lowest (mtDNA-low 5%) green fluorescent cell sub-populations. Analysis of migration activity (**A**) and invasion activity (**B**) were assessed in both sub-populations, employing a modified Boyden chamber method. Briefly, for both cell migration and invasion experiments, 24-well Transwell tissue culture inserts, with an 8μm pore size, PET membrane, were utilized. Uncoated transparent membranes were used for cell migration, while extracellular matrix pre-coated membranes were used for cell invasion. Cells were allowed 16 h for migration assays and 24 h for invasion assays. Representative images of the stained PET membranes are also shown. Three experimental repeats, each of which contains two technical replicates, were performed for each condition. Data are shown as fold change of the mtDNA-high 5% increase over mtDNA-low 5% cells. Bars represent the mean ± SD (*n* = 3). Statistical significance was determined using an unpaired Student’s *t* test, ***p ≤ 0.001.

As MDA-MB-231 cells are an established model for measuring cancer cell motility, we also assessed cell migration and invasion, using a modified Boyden chamber assay, with Transwells. Remarkably, mtDNA-high MDA-MB-231 cells showed an approximate 2-fold increase in both cell migration (Fig. 7A) and invasion (Fig. 7B). Therefore, as predicted, mtDNA-high MDA-MB-231 cells were clearly more migratory and invasive, suggesting that mtDNA content may play a key role in metastasis.

Finally, we also created a complementary genetic model in MDA-MB-231 cells to independently validate our results with SYBR Gold. Briefly, we first confirmed POLG1-silencing using a targeted shRNA approach, by Western blot analysis (Supplementary Fig. S9A, uncropped Western blot in Original Data File). Importantly, shPOLG1 cells showed decreased levels of mtDNA and reduced mammosphere formation (Supplementary Fig. S9B, C), mirroring our results with SYBR Gold mtDNA-low cells. Similarly, shPOLG1 cells showed a metabolic reduction in mitochondrial respiration and glycolytic reserve capacity (Supplementary Fig. S9E, F, G, H), with reduced cell migration and invasion levels (Supplementary Fig. S9I, J). Also, shPOLG1 cells showed decreased colony formation (Supplementary Fig. S9D), since it is a stable genetic model.

These results also provide promising evidence that POLG1 is a new potential therapeutic target for anti-cancer therapy, aimed at eradicating cancer stem cells (CSCs).

### Alovudine (3’-deoxy-3’-fluorothymidine), an established mtDNA synthesis inhibitor, prevents spontaneous cancer cell metastasis, without significant toxicity

To further investigate the potential therapeutic implications of our current findings, we selected an established mtDNA synthesis inhibitor, namely Alovudine, that targets POLG1, the mitochondrial DNA polymerase [19]

**Fig. 8:**
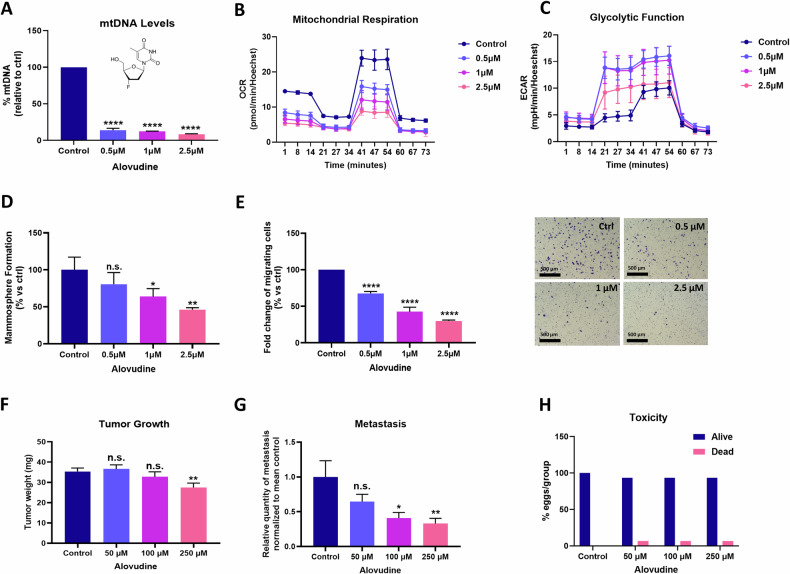
Alovudine, an established mtDNA synthesis inhibitor, induces a metabolic shift towards glycolysis and prevents spontaneous cancer cell metastasis, without significant toxicity. Alovudine-related in vitro experiments were performed after a 6-day pre-treatment of MDA-MB-231 cells. Briefly, two hundred fifty thousand cells were plated in T25 flasks and incubated for 6 days with Alovudine, at the indicated concentrations. Cells were treated at day 1 and media was renewed with Alovudine at day 4. **A** mtDNA Abundance. Relative mitochondrial DNA copy number was obtained using a Relative Human Mitochondrial DNA Copy Number Quantification qPCR Assay Kit. Data are shown as the mean ± SD (*n* = 3). Statistical significance was determined using one-way ANOVA, Dunnett’s multiple comparisons test, ****p < 0.0001. **B** Mitochondrial Function. Mitochondrial respiration was determined using the Seahorse XFe96. The graph represents the OCR (oxygen consumption rate) tracing of four experimental repeats, each of which contains five technical replicates, for each condition. **C** Glycolytic Analysis. Glycolytic function was determined using the Seahorse XFe96. The graph represents the ECAR (extracellular acidification rate) tracings of four experimental repeats, each of which contains five technical replicates, for each condition. **D** 3D Anchorage-Independent Growth. The mammosphere assay was performed in low-attachment plates for 5 days. Alovudine treatments were maintained during these 5 days. Three experimental repeats, each of which contains three technical replicates, were performed for each condition. Data are shown as mean ± SD (*n* = 3). Statistical significance was determined using one-way ANOVA, Dunnett’s multiple comparisons test, *p ≤ 0.05, **p < 0.01, n.s. not significant. **E** Cell Migration. Migration analysis was performed with uncoated PET membrane. The cells were allowed to migrate across an 8 μm pore membranes for 6 h. Three experimental repeats, each of which contains three technical replicates, were performed for each condition. Data are shown as fold change of the Alovudine treatments relative to control. Bars represent the mean ± SD (*n* = 3). Statistical significance was determined using one-way ANOVA, Dunnett’s multiple comparisons test, ****p < 0.0001. **F** Tumour Growth. Tumour weights (mg) were measured for the indicated Alovudine treatment concentrations, at the end of the CAM assay. Data are shown as the mean ± SEM (*n* = 14–15 per group). Statistical significance was determined using one-way ANOVA, Dunnett’s multiple comparisons test, **p ≤ 0.01, n.s. not significant. **G** Spontaneous Metastasis. The relative quantity of metastases in lower CAM for the indicated Alovudine treatment concentrations are shown. Data are presented as the mean ± SEM (*n* = 9–10 per group). Statistical significance was determined using one-way ANOVA, Dunnett’s multiple comparisons test, *p ≤ 0.05, n.s. not significant. **H** Embryo Toxicity. Survival of embryos for each experimental group is shown. *n* = 15 per group. Typically, in the pre-clinical animal model used here, it is recommended that one should use 100X the concentrations that were effective in cell culture assays. So, 100 times 0.5, 1, and 2.5 micromolar is 50, 100, and 250 micromolar.

As expected, treatment of MDA-MB-231 cells with Alovudine (0.5–2.5 μM) resulted in severe mtDNA-depletion, with a > 90% reduction in mtDNA levels, as evaluated by qPCR (Fig. 8A). Despite these reductions in mtDNA levels, Alovudine did not affect the viability of human fibroblasts (hTERT-BJ1) or MDA-MB-231 cancer cells, indicating that Alovudine is relatively non-toxic (Supplementary Fig. S10A,B).

However, Alovudine treatment dramatically affected the metabolic status of MDA-MB-231 cells, dose-dependently inhibiting mitochondrial respiration and resulting in mitochondrial ATP-depletion (Fig. 8B; Supplementary Fig. S10C). Conversely, Alovudine treatment induced a compensatory increase in glycolytic metabolism (Fig. 8C; Supplementary Fig. S10D). This metabolic shift, from mitochondrial to glycolytic metabolism, functionally inhibited stemness, as evidenced by dramatic decreases in 3D anchorage-independent growth, as assessed using the mammosphere assay (Fig. 8D); similar results were obtained using the colony assay, which is a well-established long-term read-out for cell proliferation (Supplementary Fig. S10E). This glycolytic shift also resulted in a dose-dependent inhibition of cell migration (Fig. 8E).

Finally, we evaluated the in vivo effects of Alovudine-induced mtDNA-depletion on tumour growth and spontaneous metastasis, using an established pre-clinical animal model, known as the CAM (chorioallantoic membrane) assay (See Supplementary Fig. S4). Briefly, implanted MDA-MB-231 tumour cell grafts were treated with Alovudine for 9 days, after which i) tumour growth, ii) spontaneous metastasis, and iii) embryo toxicity, were all quantitatively assessed. Interestingly, Alovudine only minimally inhibited tumour growth by approximately 20% (Fig. 8F), but very effectively reduced the formation of spontaneous metastases by nearly 70% (Fig. 8G). Remarkably, under these conditions, little or no embryo toxicity was observed (Fig. 8H).

In summary, drug-induced mtDNA-depletion resulted in a dose-dependent shift, from mitochondrial to glycolytic metabolism. Functionally, this metabolic shift towards glycolysis resulted in a loss of stemness, inhibition of cell migration, and prevention of spontaneous metastasis, without significant toxic effects. In contrast, tumour growth remained largely unaffected. Therefore, POLG1-driven mtDNA synthesis in CSCs may represent a new promising mitochondrial target for the therapeutic prevention of cancer cell metastasis.

## Discussion

Here, we investigated possible links between mitochondrial DNA (mtDNA) levels and cancer stemness. Interestingly, several previous studies have clinically assessed the potential role of mtDNA copy number in the pathogenesis of human breast cancer. For example, high mtDNA copy number increases the risk of developing breast cancer [27–29], by up to 6.4-fold [28], likely secondary to increased mitochondrial oxidative stress. Similarly, in patients with breast cancer metastasis, the primary tumour tissue showed a significant increase in mtDNA copy number by ≥2-fold, relative to matched adjacent non-tumour tissue and blood levels [30]. In other cohorts, high mtDNA levels were associated with increased breast tumour size (>2-cm), increased tumour grade (especially grade 3), ER-negative status, and a lack of response to anthracycline-based chemotherapy (OR (95% CI) = 2.48 (0.97–6.61)) [31, 32]. Conversely, breast tumours with low mtDNA copy number were associated with longer progression-free survival (PFS), in both univariate and multivariate analysis [31]. Also, increased mtDNA copy number facilitated cell proliferation and metastasis in colorectal cancer [33]. Additionally, an increase in mtDNA and mitochondrial biogenesis was reported in endometrial carcinoma [34].

In our current studies, we observed an increase in stemness features in cells enriched with high levels of mtDNA, such as 3D anchorage-independent growth, mitochondrial metabolic activity, and CD44 levels, as well as drug-resistance to Gemcitabine and Tamoxifen. We used the SYBR Gold dye at low concentrations to selectively stain mtDNA, as reported previously [18]. Our flow cytometry approach permitted the collection of two cell sub-populations, according to their mtDNA levels, i.e., mtDNA-high cells and mtDNA-low cells. As predicted, SYBR Gold-stained mitochondrial nucleoids were clearly more abundant in the mtDNA-high MCF7 sub-population. To independently validate the model, we used a specific DNA-binding mAb probe, namely AC-30-10. This antibody has been used in many studies to specifically stain mtDNA [21, 22]. AC-30-10 mAb-staining revealed the same characteristic mitochondrial nucleoid pattern, especially in mtDNA-high MCF7 cells. Moreover, mtDNA encodes 13 proteins involved in oxidative phosphorylation (OXPHOS), including MT-CO1, MT-CO2, MT-CO3, MT-CYB or MT-ND2 [35]. All of these 5 gene transcripts or proteins were increased in the mtDNA-high sub-population, as assessed by RT-qPCR, immuno-staining, and/or FACS analysis.

As discussed above, MT-CO2 immuno-staining is a tightly-linked surrogate marker of mtDNA content. In accordance with our current findings, MT-CO2 is also a clinically-validated protein biomarker for metastasis, in large cohorts of both breast and prostate cancer patients [36, 37]. In addition, in this breast cancer cohort (*N* = 2197 patients), high MT-CO2 protein expression was associated with elevated Ki67 (a marker of cell proliferation) [36]. Importantly, higher levels of MT-CO2 immuno-staining were observed in breast cancer tissue samples, as compared with normal breast tissue. These findings suggest that neoplastic transformation may require higher levels of mtDNA, functionally driving more active mitochondrial ATP-production. Increased MT-CO2 immuno-staining in breast cancer primary tumour samples also correlates with rising tumour grade and stage [36]. Similarly, MT-CO2 expression is a strong predictor of poor clinical outcome in prostate cancer (*N* = 11,152 patients) [37]. As such, our current mechanistic studies provide a molecular explanation for these clinical findings. Here, our analysis of MT-CO2 (+) mtDNA-high cells revealed increased stemness features, as well as functionally more active mitochondria, with increased mitochondrial respiration and ATP production. Similarly, we previously observed that MT-CO2 is over-expressed in an hTERT-enriched sub-population of MCF7 breast cancer stem cells [38].

In yeast cells, mtDNA controls cell cycle progression [39–42]. Several studies have shown that the mtDNA copy number is increased in growing cultures of yeast cells in the respiratory phase and are lower in the fermentation phase. In fact, some yeast species, called rho-negative cells, could survive without mtDNA under conditions of fermentation, but they are characterised by slow growth on fermentable carbon sources and an inability to grow on non-fermentable sources [39–42]. Similarly, here we observed that mtDNA-high MCF7 cells exhibited significantly increased proliferation rates, with a dramatic shift towards the S- and G2/M-phases of the cell cycle. These results were independently validated by RNA-Seq analysis, showing enrichment of cell cycle-regulated gene transcripts, especially those related to the G2/M phase, nuclear division and chromosomal segregation. Therefore, high mtDNA levels may also increase proliferation rates in cancer cells.

Complementary results were obtained with mtDNA-high MDA-MB-231 cells, which exhibited increased stemness features, higher proliferation rates, as well as increased cell migration and invasion, suggesting a role for mtDNA in metastasis. Several studies confirm the relation between the mtDNA mutations and cancer cell metastasis [43]. For example, Ishikawa et al. demonstrated that replacement of mtDNA in a non-metastatic cell line, with mtDNA from a highly metastatic cell line was indeed sufficient to confer metastatic behaviour in vivo [44].

Furthermore, we also created a genetic model to independently validate our results, by silencing POLG1 gene expression, using a targeted shRNA approach. As predicted, genetic ablation of POLG1 expression dramatically reduced mtDNA levels, and mirrored the phenotypes that we observed by cell sorting with SYBR Gold. Similarly, in our current studies, inhibition of mtDNA synthesis with Alovudine was sufficient to deplete mtDNA by >90% and prevent spontaneous metastatic dissemination.

[^18^F]-FLT (Fluoro-thymidine F-18; CAS: 287114-80-1) is a chemical entity that is identical to Alovudine (CAS: 25526-93-6), with the exception that the fluorine atom is a radioactive isotope that can be visualized by PET (Positron Emission Tomography). Because it is used as a radioactive tracer for PET imaging, [^18^F]-FLT is used in trace amounts, very low concentrations that would not be sufficient to be therapeutically active. Clinically, higher [^18^F]-FLT signal intensity is specifically associated with highly proliferative [45, 46] and metastatic cancer cells [47, 48], as well as poor clinical outcomes in cancer patients [49]. Unfortunately, the reasons for these strong clinical associations were not mechanistically understood, or previously linked to mitochondria.

However, in 2019, higher concentrations of Alovudine (200 nM to 2 μM) were shown for the first time to induce mtDNA-depletion, by acting as a specific inhibitor of POLG1, the mitochondrial DNA polymerase [19]. Taken together, these data directly imply that [^18^F]-FLT labels highly proliferative and metastatic cancer cells in vivo, because they have increased levels of mtDNA synthesis and/or higher mtDNA content.

In accordance with this hypothesis, here we show that cancer cells with higher mtDNA content are indeed hyper-proliferative, and that mtDNA-depletion with Alovudine effectively targets proliferative CSCs, preventing metastasis in a preclinical animal model of tumourigenesis. Therefore, in the future, [^18^F]-FLT PET imaging may be a useful tool for clinically monitoring the efficacy of Alovudine for therapeutically targeting and eliminating hyper-proliferative/metastatic CSCs in patients. This would allow a real-time and dynamic assessment of the response to therapy with Alovudine, or other related POLG1-inhibitors.

Finally, our current findings with Alovudine treatment are consistent with a recent non-peer reviewed pre-print appearing in *bioRxiv* [50]. Briefly, these investigators showed that pathogenic mtDNA mutations, which block OXPHOS and mitochondrial ATP-production, can effectively inhibit melanoma metastasis in vivo, but have only minor effects on primary tumour growth. These results indicate that metastasis is dependent on functional mtDNA [50]. Similarly, we have previously shown that metastasis is strictly dependent on i) mitochondrial ribosome function, and ii) sufficient levels of mitochondrially-derived ATP, using two FDA-approved drugs, namely Doxycycline and Bedaquiline [23, 24, 51, 52]. Doxycycline is a specific inhibitor of mitochondrial protein synthesis, while Bedaquiline targets the gamma-subunit (ATP5F1C) of the mitochondrial ATP synthase, preventing ATP production [23, 24, 51, 52]. Taken together, these findings suggest a druggable, linear, mitochondrial biosynthetic pathway (mtDNA → mitochondrial protein synthesis → ATP production) [Supplementary Fig. S11], for mechanistically understanding and preventing metastatic disease progression in breast cancers, and possibly other cancer types.

## Supplementary information

Supplemental Information

Supplemental Figures

Original Data File

## Data Availability

The original contributions presented in the study are included in the article. Further inquiries can be directed to the corresponding authors.
