# Supplementary material for: High mitochondrial DNA content is a key determinant of stemness, proliferation, cell migration, and cancer metastasis in vivo
Source: Cell Death Dis. 2024 Oct 11;15(10):745. doi: 10.1038/s41419-024-07103-9 (PMC11470112; doi:10.1038/s41419-024-07103-9)
Supplement: Supplementary file 1 — Supplemental Information [file 41419_2024_7103_MOESM1_ESM.pdf]

Supplemental information: Material and Methods

BioTracker ATP-Red Live Cell Dye: Staining and Cell Isolation

MCF7 and MDA-MB231 cells were stained with BioTracker ATP-Red 1 (Sigma-Aldrich, # SCT045). After incubation cells were subjected to FACS sorting, using the SONY SH800 Cell Sorter, to isolate the 5% highest ATP (ATP-high 5%) and the 5% lowest ATP (ATP-low 5%) subpopulations. These sub-populations were subjected to colony assay.

Cell Viability Assay

The Sulphorhodamine (SRB) assay was performed in 96-well plates. After 72 hours of Alovudine treatments at the indicated concentrations, cells were fixed with 10% trichloroacetic acid (TCA) for 1h in the cold room (4°C) and were dried overnight at room temperature. Then, cells were incubated with SRB for 15 min, washed twice with 1% acetic acid, and air dried for at least 1h. The protein-bound dye was dissolved in a 10 mM Tris, pH 8.8, solution. Finally, protein content was evaluated using the Varioskan ™ LUX plate reader (ThermoFisher Scientific) reading the plate at 540-nm.

Colony Formation Assay

Two hundred fifty cells were seeded per well in 6-wells-plates and incubated for 14 days after sorting, or treated with Alovudine, at indicated concentrations. Cells were treated at day 1 and media was renewed with Alovudine at day 7. Colonies were fixed with ethanol 70% for 30 mins, stained in 0.5% crystal violet for 30 minutes, rinsed in water and examined under a bright-field microscope.

RNA Extraction

RNA was extracted using the Monarch Total RNA Miniprep Kit (New England Biolabs, #T2010S), following manufacturer's instructions for cultured mammalian cells, and samples were diluted to a final concentration of 20 ng/µl.

RNA Sequencing Analysis

Total RNA was purified using Monarch® Total RNA Miniprep Kit (New England Biolabs) following manufacturer's instructions (n = 4 per experimental condition). Novogene's (Novogene (UK) Company Limited, Cambridge, UK) RNA sequencing services were used for all mRNA sequencing. Briefly, an mRNA library was generated from total RNA, quantified, and sequenced using the Illumina NovaSeq 6000 platform (Illumina, San Diego, CA). Clean data was obtained from raw reads, and all downstream analysis were based on clean data. Reference genome index was built using Hisat2 v2.0.5. Differential expression analysis was completed using DESeq2, and P-values were adjusted using the Benjamini and Hochberg's approach for controlling the false discovery rate. Genes with an adjusted P-value <=0.05 found by DESeq2 were assigned as differentially expressed. Enrichment analyses were completed using the clusterProfiler R package to test the statistical enrichment of differential expression genes in GO, KEGG and Reactome databases. RNAseq data will be publicly accessible in the Harvard Dataverse repository upon publication (RNAseq data files: Michael Lisanti, 2024, "RNAseq data from MCF-7 cells selected for high and low SYBR-Gold staining intensity.", https://doi.org/10.7910/DVN/WVNTLH, Harvard Dataverse).

Cell death analysis

Analysis of the cell death was performed using the Muse® Annexin V & Death Cell Kit (Luminex, #MCH100105). After sorting, cells were incubated with Annexin V and 7-AAD for 20 min and analysed by flow cytometry (Attune ™ NxT Flow Cytometer, ThermoFisher Scientific).

Western Blotting

Cells were lysed in RIPA buffer (Sigma-Aldrich, #R0278), containing protease and phosphatase inhibitors (Roche, #04906845001 #05892970001). After protein quantification by BCA protein assay kit, total lysates were loaded onto SDS-polyacrylamide gels (SDS-PAGE; Mini-Protean TGX Gel, 4-20%, Bio-Rad, # 4561094). The gels were transferred onto 0.2-µm nitrocellulose membranes (Mini Trans-Blot Turbo Transfer Pack, Bio-Rad, #1704158), using the TransBlot Turbo Transfer System (Bio-Rad). Membranes were blocked with 5% BSA PBS-T (PBS 1%; 0.5% Tween 20, Sigma-Aldrich, #) for 1 hour at room temperature in an orbital shaker. Subsequently, the membranes were incubated with primary antibodies in 5% BSA/PBS-T for

12–16 h at 4°C, followed by incubation with fluorescence secondary antibodies (LI-COR; IRDye® 680RD Donkey anti-Rabbit IgG, #925-68073; IRDye® 800CW Goat anti-Mouse IgG, #925-32210) for 1 h at room temperature. Antibodies against the following proteins were used: POLG1 (Abcam, #97661) and β-Tubulin (Santa Cruz Biotechnology, #SC-5274). β-Tubulin was used as protein loading control. The resulting images were acquired using Odyssey XF (LI-COR).

Viral Transduction and Cell Selection

Lentiviral constructs from GeneCopoeia (shPOLG1 Clone C, #CS-HSH063505-LVRU6GP and shCONTROL, #CSHCTR001-LVRU6GP) were amplified and used to stably-transduce MDA-MB-231 cells. After cell transduction, the cell lines were selected with puromycin for two weeks. After selection, both cell lines were subjected to different functional assays, to characterize their phenotypic differences experimentally. Both cell lines were selected and maintained in the presence of Uridine (0.05mg/ml, Sigma-Aldrich, #U3003).

Legends for Supplemental Figures, namely S1 to S11.

Supplemental Figure S1. Experimental Strategy for Cell Sorting with SYBR Gold. Here, we investigated the relationship between mitochondrial DNA (mtDNA) content and aggressive cancer cell phenotypes. For this purpose, we used SYBR Gold, a nucleic acid dye, to obtain two cell sub-populations, based on its green fluorescence intensity, using flow cytometry. More specifically, we stained MCF7 cells with SYBR Gold for 30 minutes at a dilution of 1:20,000. Previous studies reported that low concentrations of SYBR Gold only stained mitochondrial DNA. After performing flow cytometry with the SONY SH800 Cell Sorter, we obtained two cell sub-populations: i) one with high levels of mtDNA (mtDNA-high 5%) and ii) another with low levels of mtDNA (mtDNA-low 5%). The chemical structure of SYBR Gold is also shown, which is a lipophilic cation (a known mitochondrial-targeting signal).

Supplemental Figure S2. RT-qPCR analyses of mtDNA-high and mtDNA-low cell sub-populations. Primer pairs for amplifying four mtDNA-encoded transcripts are shown: MT-CYB, MT-CO1, MT-ND2 and MT-CO3. Primer pairs for the two reference transcripts (for normalisation), are also included: GAPDH and UBC. (A) Sequences of primer pairs used for RT-qPCR; (B) Melt curve analysis of primer pairs used for RT-qPCR.

Supplemental Figure S3. Quantitation of Immuno-fluorescence Staining. (A) Merged images of MT-CO1 and MT-CO2 immuno-staining. (B) ImageJ macro used to quantify immune-fluorescence staining. (C) Image analysis workflow for immune-staining. Merged images were split into individual channels. Nuclei were segmented and counted using a StarDist2.0 plugin (1). Background was removed by thresholding, converting to a mask, and applying it to the original channel image, before measuring the mean grey intensity, and calculating mean grey intensity per cell.

Supplemental Figure S4. Experimental Approach for Evaluating the *In Vivo* Efficacy of Alovudine: Tumour Growth, Spontaneous Metastasis, and Drug Toxicity.

Fertilized White Leghorn eggs were incubated at 37.5°C with 50% relative humidity for 9 days. On embryonic day 9 (E9), the chorio-allantoic-membrane (CAM) was dropped down by drilling a small hole through the eggshell into the air sac, and a 1 cm^2 window was cut in the eggshell above the CAM. The MDA-MB-231 tumour cell line was cultivated in DMEM medium supplemented with 10% FBS and 1% penicillin/streptomycin. On day E9, MDA-MB-231 cells were detached with trypsin, washed with complete medium and suspended in graft medium. An inoculum of 1 X 10^6 cells was added onto the upper CAM of each egg (E9) and then eggs were randomized into groups. On day E10, tumours were detectable, and they were then treated daily for 9 days with vehicle alone (0.5% DMSO in PBS) or with three different dosages of Alovudine (50, 100 and 250 μM). At day embryonic day 18 (E18), the upper portion of the CAM was removed from each egg, washed in PBS and then directly transferred to paraformaldehyde (fixation for 48 h) and weighed. For tumour growth assays, at least 14-15 tumour samples were collected and analysed per group (n = 14-15). On day E18, a 1 cm^2 portion of the lower CAM was collected to evaluate the number of metastatic cells in 9-10 samples per group (n = 9-10). Genomic DNA was extracted from the CAM (using a commercial kit) and analysed by qPCR with specific primers for Human Alu sequences. Calculation of Cq for each sample, mean Cq and relative amounts of metastases for each group are directly calculated using the Bio-Rad® CFX Maestro software. Embryo tolerability (drug toxicity) was evaluated by scoring the number of live and dead embryos, on day E18, at the end of the experiment.

Supplemental Figure S5. Assessment of cell death in MCF7 and MDA-MB-231 sub-populations, after sorting by SYBR Gold dye. MCF7 and MDA-MB-231 cells were incubated for 30 minutes with low concentrations of SYBR Gold dye (at a dilution of 1:20,000). After cell staining, samples were subjected to FACS sorting to isolate the 5% highest (mtDNA-high 5%) and the 5% lowest (mtDNA-low 5%) green fluorescent cell sub-populations**.** Cell death was then determined by Annexin V and 7-ADD staining, in MCF7 (A) and MDA-MB231 (B). Graphs show a representative experiment: Annexin V (-)/7-ADD (-) represents live cells, Annexin V

(+)/7-ADD (-) early stages of apoptosis, Annexin V (+)/7-ADD (+) late stages of apoptosis and Annexin V (-)/7-ADD (+) dead cells. The percentages of each stage are included in the panels. Note that the vast majority of the cells are indeed alive and do not undergo cell death.

Supplemental Figure S6. Representative FACS plots for CD44 staining and cell cycle analysis.

(A) Representative plot for CD44-APC antibody and isotype antibody control-APC (IgG-APC).
(B) Representative plot for cell cycle analysis.

Supplemental Figure S7. Colony Formation Assays do not show Differences, after Sorting Using either SYBR Gold or BioTracker ATP-Red 1. MCF7 and MDA-MB-231 cells were stained with SYBR Gold, and subjected to flow cytometry, with the SONY SH800 Cell Sorter. Then, after isolation of the i) mtDNA-high 5% and ii) the mtDNA-low 5% sub-populations, the cells were seeded for colony formation assays. Colonies were counted after 14 days of culture. For comparison purposes, cells were also independently stained with BioTracker ATP-Red 1, as high ATP levels are a known biomarker of stemness. Four experimental repeats, each of which contains three technical replicates, were performed for each condition. Data are shown as the mean ± SD (n = 4), representing the mtDNA-high 5% increase over mtDNA-low 5% cells, or the ATP-high 5% increase over ATP-low 5%. Statistical significance was determined using an unpaired Student's t-test, n.s. not significant.

Supplemental Figure S8. Enrichment of Cell Cycle Related mRNA Transcripts in mtDNA-high MCF7 cells. (A) Resulting Volcano Plots are shown. Note that 520 transcripts were up-regulated, and 390 were down-regulated, while 24,239 remained unchanged, when comparing mtDNA-high MCF7 cells, relative to mtDNA-low MCF7 cells. (B-D). Bioinformatics Analysis. Gene set enrichment analysis (GSEA) indicated significant enrichment of cell cycle progression, the G2/M phase, nuclear division, and chromosomal segregation related genes, using the KEGG, Reactome, and the GO databases, in mtDNA-high MCF7 cells, relative to mtDNA-low MCF7 cells. This is consistent with phenotypic data showing an increase in

proliferation and cell cycle progression in mtDNA-high MCF7 cells. See **Figure 3 (panels D and E)**, within the main text, for functional evidence.

Supplemental Figure S9. A genetic model of mtDNA depletion, using POLG1-targeted shRNA, shows decreased mammosphere formation and reduced colony formation, as well as inhibition of mitochondrial respiration, cell migration and invasion. MDA-MB-231 cells were transfected with shCONTROL and shPOLG1 constructs. After two-weeks selection, both cell lines were subjected to different functional assays, to characterize their phenotypic differences experimentally.

(A) Western blot. POLG1 protein levels were determined by Western blotting in shCONTROL and shPOLG1 cell lines. β-Tubulin was used as a protein loading control (uncropped original western blot in **Original Data File**).

(B) mtDNA Abundance. Relative mitochondrial DNA copy number was obtained using a Relative Human Mitochondrial DNA Copy Number Quantification qPCR Assay Kit. Data are shown as the mean ± SD (n = 3). Bars represent the mean ± SD (n=3). Statistical significance was determined using an unpaired Student's t-test, ****p < 0.0001.

(C) 3D Anchorage-Independent Growth. The mammosphere assay was performed in low-attachment plates for 5 days. Data are shown as mean ± SD (n = 3). Statistical significance was determined using an unpaired Student's t-test, **p < 0.01.

(D) Colony Formation Assay. Colony assay was performed for two weeks. Bar graphs in the **Higher panel** show the mean of three experimental repeats, each of which contains three technical replicates, for each condition. The **Lower panel** shows images of a representative experiment. Statistical significance was determined using an unpaired Student's t-test, ****p < 0.0001.

(E) Mitochondrial Function. Mitochondrial respiration was determined using the Seahorse XFe96. The graph represents the OCR (oxygen consumption rate) tracing of four experimental repeats, each of which contains three technical replicates, for each condition.

(F) Mitochondrial Function Parameters. Bars graphs illustrate basal respiration, proton leak, maximal respiration, spare respiratory capacity, and ATP production, obtained from the OCR quantification by Seahorse XFe96. Four experimental repeats, each of which contains three technical replicates, were performed for each condition. Data are shown as mean ± SD (n = 4). Statistical significance was determined using an unpaired Student's t-test, * $p < 0.05$, **$p < 0.01$.

(G) Glycolytic Analysis. Glycolytic function was determined using the Seahorse XFe96. The graph represents the ECAR (extracellular acidification rate) tracings of four experimental repeats, each of which contains three technical replicates, for each condition.

(H) Glycolytic Analysis Parameters. Bars graphs show glycolysis, glycolytic capacity and glycolytic reserve obtained from the ECAR quantification by Seahorse XFe96. Four experimental repeats, each of which contains three technical replicates, were performed for each condition. Data are shown as mean ± SD (n = 4). Statistical significance was determined using an unpaired Student's t-test, *$p < 0.05$, n.s. not significant.

(I) Cell Migration. Migration analysis was performed with uncoated PET membrane. The cells were allowed to migrate across an 8µm pore membranes for 6 hours. Three experimental repeats, each of which contains three technical replicates, were performed for each condition. Data are shown as fold change of the shPOLG1 treatments relative to shCONTROL. Data are shown as mean ± SD (n = 3). Statistical significance was determined using an unpaired Student's t-test, ***$p < 0.001$. Representative images of the stained PET membranes are also shown.

(J) Cell Invasion. Invasion analysis was performed with coated PET membrane. The cells were allowed to migrate across an 8µm pore membranes for 6 hours. Three experimental repeats, each of which contains three technical replicates, were performed for each condition. Data are shown as fold change of the shPOLG1 treatments relative to shCONTROL. Data are shown as mean ± SD (n = 3). Statistical significance was determined using an unpaired Student's t-test, ****$p < 0.0001$. Representative images of the stained PET membranes are also shown.

Supplemental Figure S10. Alovudine Treatment of MDA-MB-231 cells is Non-Toxic, but Induces a Glycolytic Shift in Cell Metabolism, and Inhibits Colony Formation.

(A) Viability in Alovudine-treated normal human fibroblasts. The effects of Alovudine on hTERT-BJ1 cell viability were assessed at the indicated concentrations, after three days of treatment, using the SRB assay. Three experimental repeats, each of which contains six technical replicates, were performed for each condition. Data are shown as mean ± SD (n = 3). Statistical significance was determined using one-way ANOVA, Dunnett's multiple comparisons test, n.s. not significant.

(B) Viability in Alovudine-treated MDA-MB-231 cells. The effects of Alovudine on MDA-MB-231 cell viability were assessed at the indicated concentrations, after three days of treatment, using the SRB assay. Three experimental repeats, each of which contains six technical replicates, were performed for each condition. Data are shown as mean ± SD (n = 3). Statistical significance was determined using one-way ANOVA, Dunnett's multiple comparisons test, n.s. not significant.

(C) Mitochondrial Function in Alovudine-treated MDA-MB-231 cells. Bars graphs illustrate basal respiration, proton leak, maximal respiration, spare respiratory capacity, and ATP production, obtained from the OCR quantification by Seahorse XFe96. Four experimental repeats, each of which contains five technical replicates, were performed for each condition. Data are shown as the mean ± SD (n = 4). Statistical significance was determined using one-way ANOVA, Dunnett's multiple comparisons test, $^{**}p < 0.01$, $^{***}p < 0.001$, $^{****}p < 0.0001$, n.s. not significant.

(D) Glycolytic Function in Alovudine-treated MDA-MB-231 cells. Bars graphs show glycolysis, glycolytic capacity and glycolytic reserve obtained from the ECAR quantification by Seahorse XFe96. Four experimental repeats, each of which contains five technical replicates, were performed for each condition. Data are shown as mean ± SD (n = 4). Statistical significance was determined using one-way ANOVA, Dunnett's multiple comparisons test, $^{*}p \leq 0.05$, $^{***}p < 0.001$, $^{****}p < 0.0001$, n.s. not significant.

(E) Colony Formation Assays with Alovudine-treated MDA-MB-231 cells. Colony assays were carried out for two weeks, at the indicated Alovudine concentrations. Alovudine treatments

commenced on day 1, after plating, and fresh media with Alovudine was added on day 8. Bar graphs in the **Left panel** show the mean of three experimental repeats, each of which contains three technical replicates, for each condition. The **Right panel** shows images of a representative experiment, at the indicated concentrations. Data are shown as the mean ± SD (n = 3). Statistical significance was determined using one-way ANOVA, Dunnett's multiple comparisons test, ***p < 0.001, ****p < 0.0001.

Supplemental Figure S11. Mitochondrial Biosynthetic Pathway. Note that all three drugs inhibit mitochondrial biogenesis, ATP production, and cancer metastasis.

Supplemental References (See also the Legend of Supplemental Figure S3)

(1) Schmidt U, Weigert M, Broaddus C, Myers G. Cell Detection with Star-Convex Polygons. In: Medical Image Computing and Computer Assisted Intervention – MICCAI 2018 Lecture Notes in Computer Science. 2018. p. 265–73.
