# Supplementary material for: High mitochondrial DNA content is a key determinant of stemness, proliferation, cell migration, and cancer metastasis in vivo
Source: Cell Death Dis. 2024 Oct 11;15(10):745. doi: 10.1038/s41419-024-07103-9 (PMC11470112; doi:10.1038/s41419-024-07103-9)

Supplemental Figure S1

Experimental Strategy

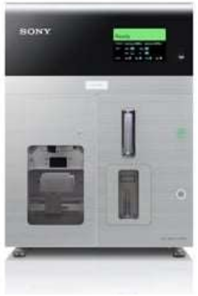

30min incubation

MCF7 → **SYBR Gold 1:20,000 dilution** → **SORTER**

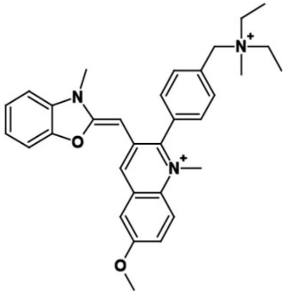

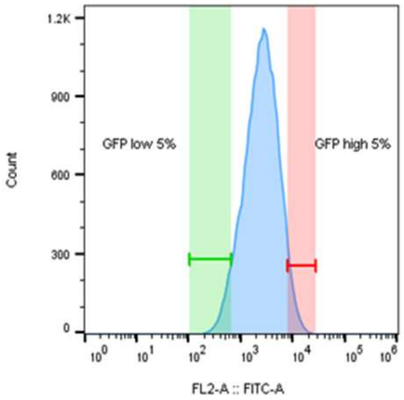

Supplemental Figure S2

A

Gene	Forward primer (5' – 3')	Reverse primer (5' – 3')
MT- CYB	TCATCGACCTCCCCACCCCATC	CGTCTCGAGTGATGTGGGCGATT
MT-CO1	CTGCTATAGTGGAGGCCGGA	GGGTGGGAGTAGTTCCCTGC
MT-CO3	CCAATGATGGCGCGATG	CTTTTTGGACAGGTGGTGTGTG
MT-ND2	CACAGAAGCTGCCATCAAGTA	CCGGAGAGTATATTGTTGAAGAG
GAPDH	CCCACTAACATCAAATGGGG	CCTTCCACAATGCCAAAGTT
UBC	CATGTACGTTGCTATCCAGGC	CTCCTTAATGTCACGCACGAT

B

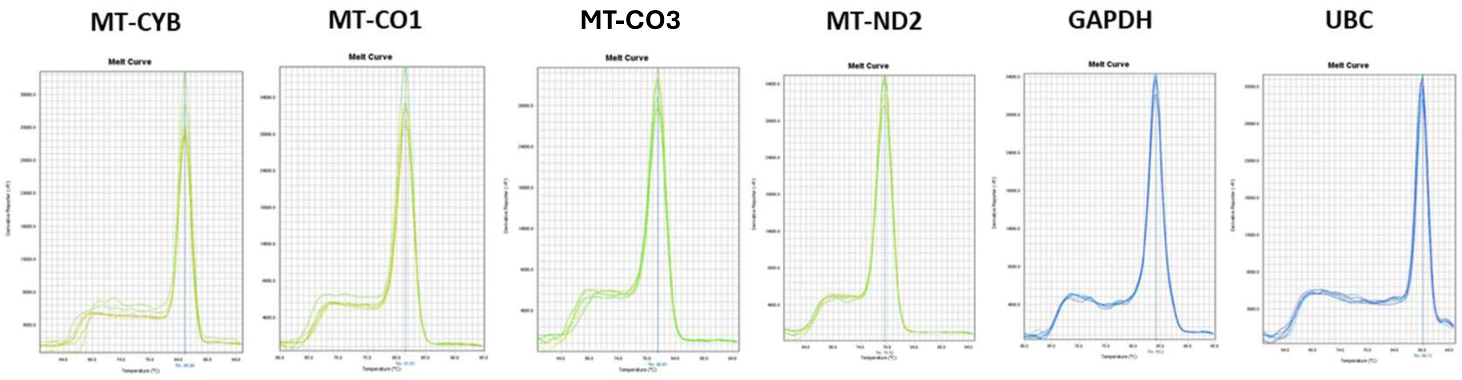

Supplemental Figure S3

A

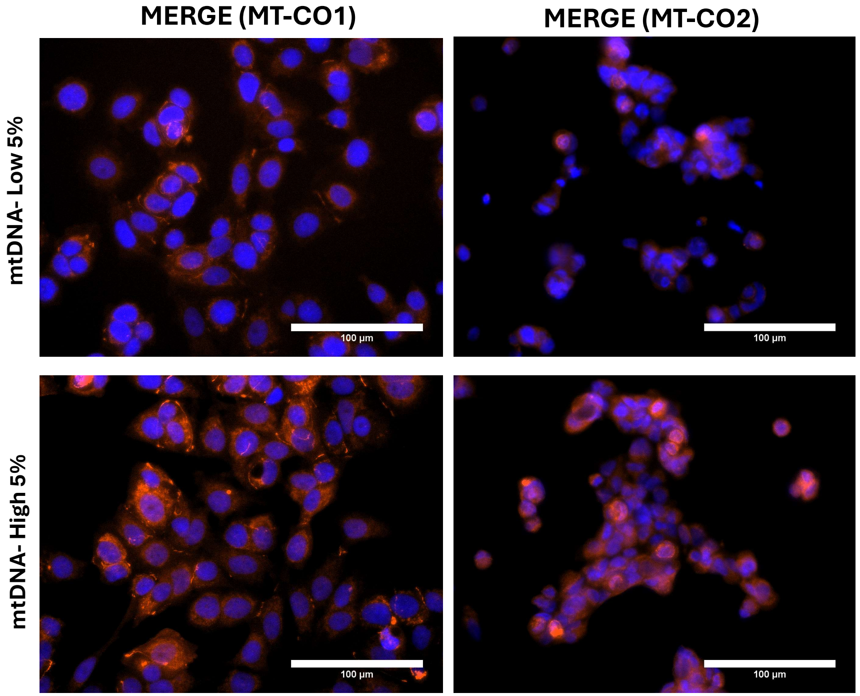

B

```
rename("ORIGINAL");
run("Duplicate...", "title=[WORKING IMAGE]");
run("Split Channels");
selectWindow("WORKING IMAGE (blue)");
run("Command From Macro", "command=[de.csbdresden.stardist.StarDist2D],
args=['input':'WORKING IMAGE (blue)', 'modelChoice':'Versatile (fluorescent
nuclei)', 'normalizeInput':'true', 'percentileBottom':'1.0',
'percentileTop':'99.8', 'probThresh':'0.5', 'nmsThresh':'0.4',
'outputType':'Both', 'nTiles':'1', 'excludeBoundary':'2',
'roiPosition':'Automatic', 'verbose':'false', 'showCsbdeepProgress':'false',
'showProbAndDist':'false'], process=[false]");
selectWindow("Label Image");
close();
selectWindow("WORKING IMAGE (blue)");
close();
selectWindow("WORKING IMAGE (green)");
close();
selectWindow("WORKING IMAGE (red)");
run("Duplicate...", " ");
selectWindow("WORKING IMAGE (red)-1");
setAutoThreshold("Default dark no-reset");
setThreshold(50, 255, "raw");
setOption("BlackBackground", true);
run("Convert to Mask");
selectWindow("WORKING IMAGE (red)");
imageCalculator("AND create", "WORKING IMAGE (red)","WORKING IMAGE (red)-1");
selectWindow("Result of WORKING IMAGE (red)");
run("Measure");
roiManager("List");
selectWindow("WORKING IMAGE (red)");
close();
selectWindow("WORKING IMAGE (red)-1");
close();
selectWindow("ORIGINAL")
close();
selectWindow("Result of WORKING IMAGE (red)");
close();
```

C

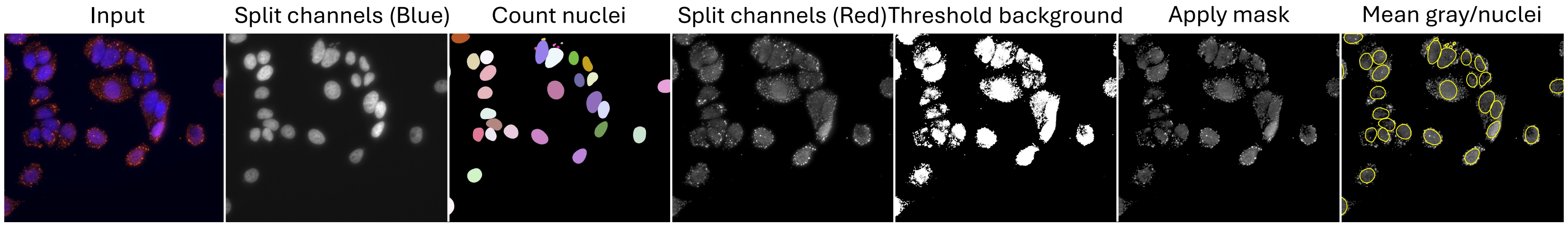

Supplemental Figure S4

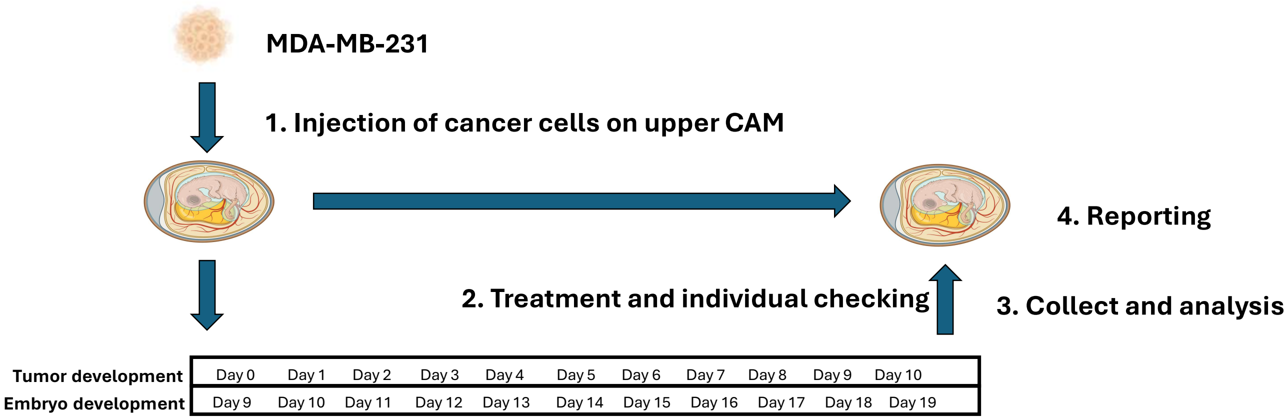

Group description	Compound name	Concentration [µM]
1	Ctrl	-
2	Alovudine	50
3	Alovudine	100
4	Alovudine	250

Supplemental Figure S5

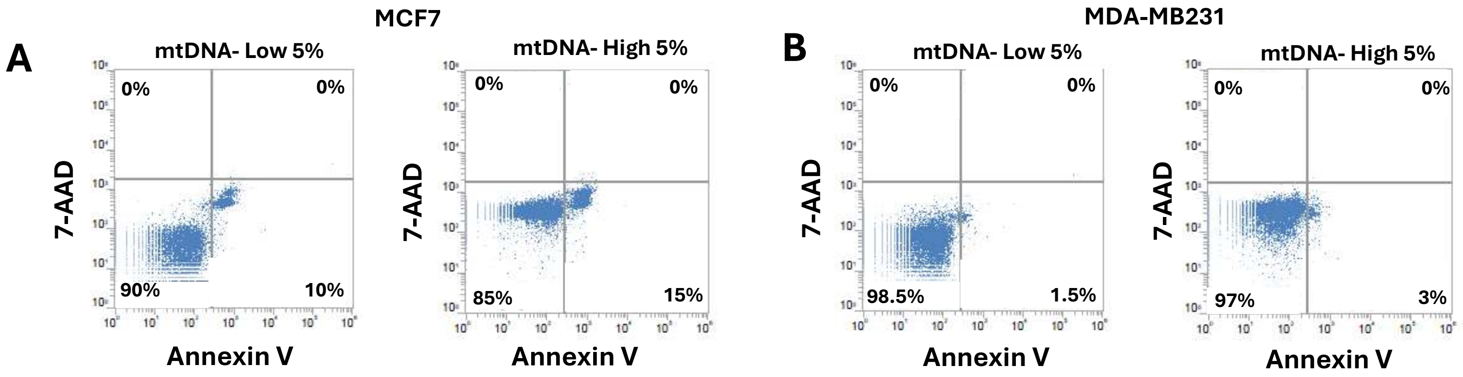

Supplemental Figure S6

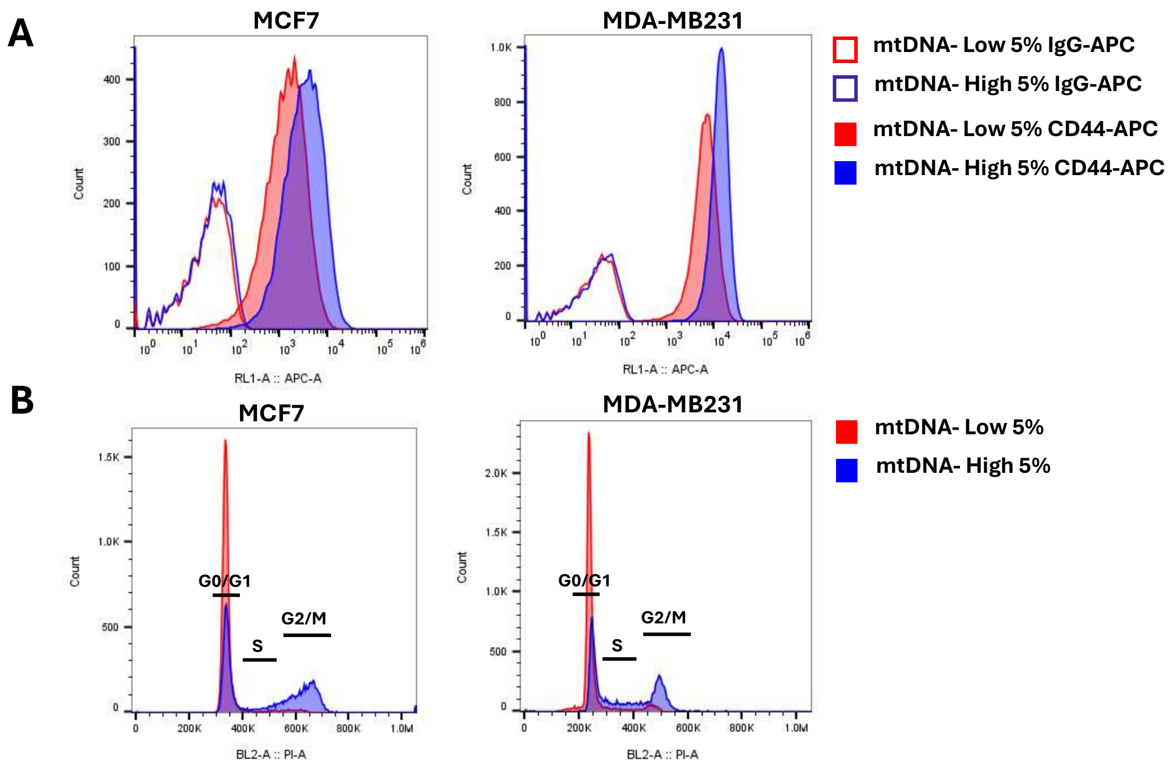

Supplemental Figure S7

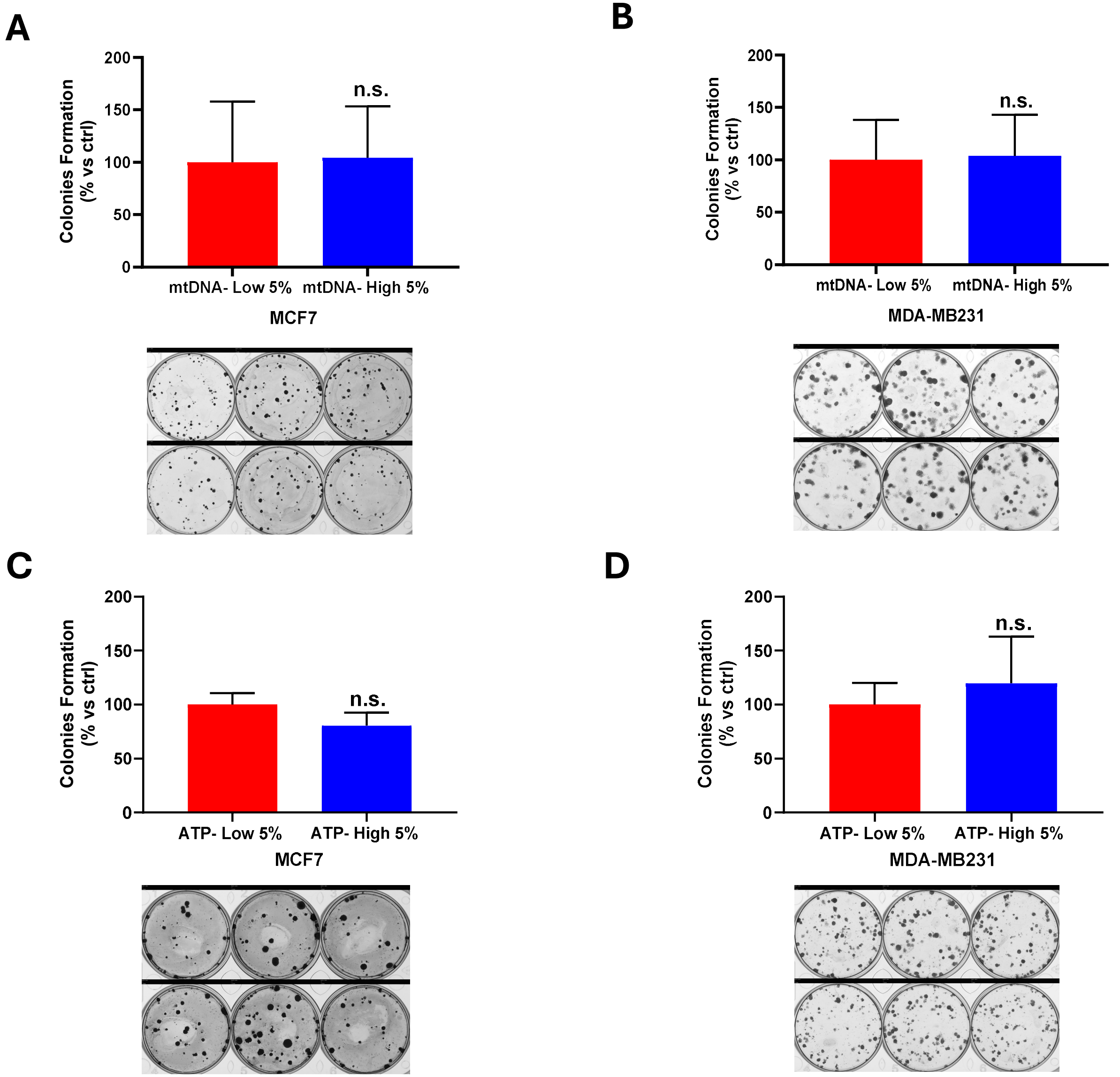

Supplemental Figure S8

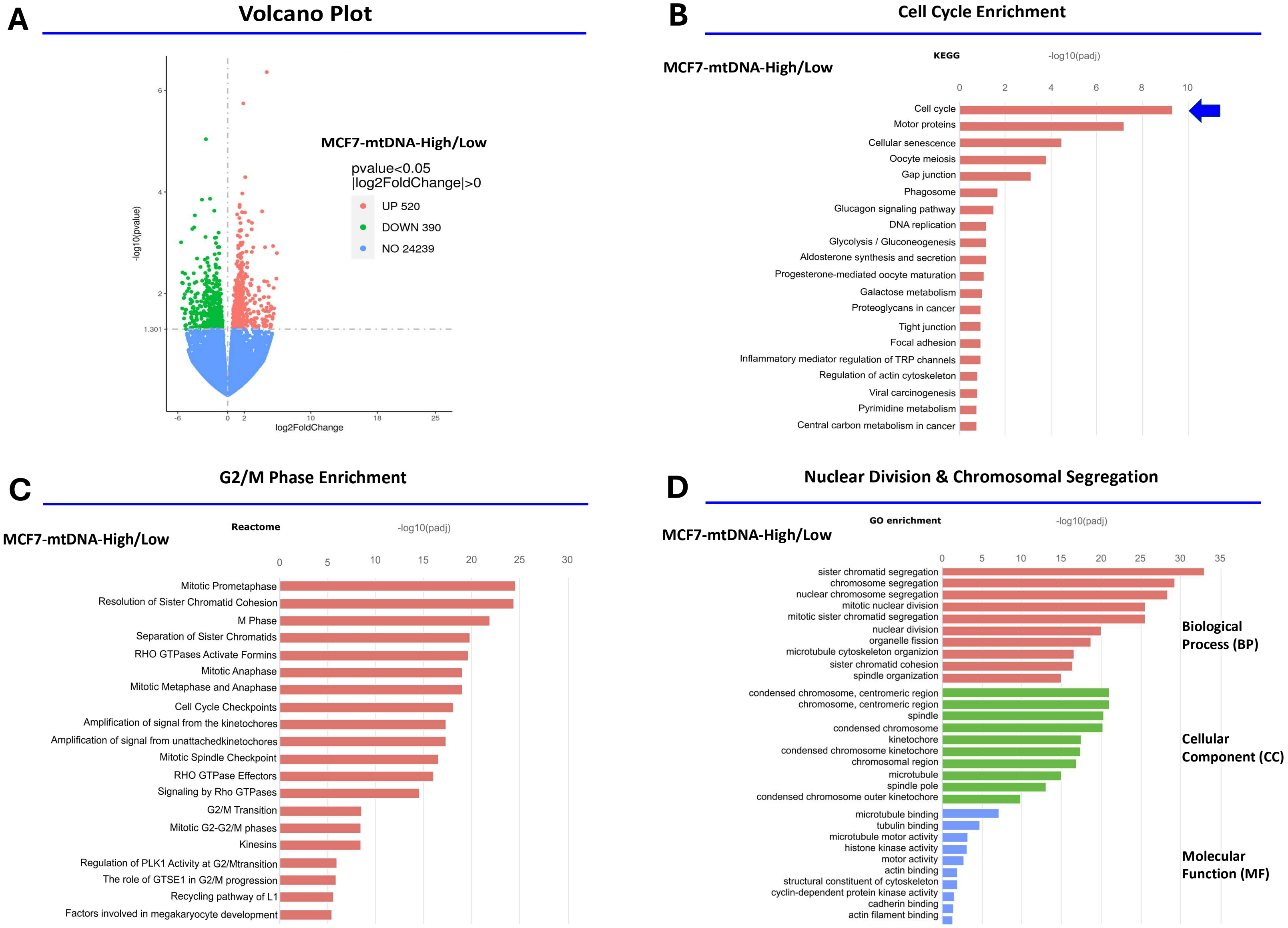

Supplemental Figure S9

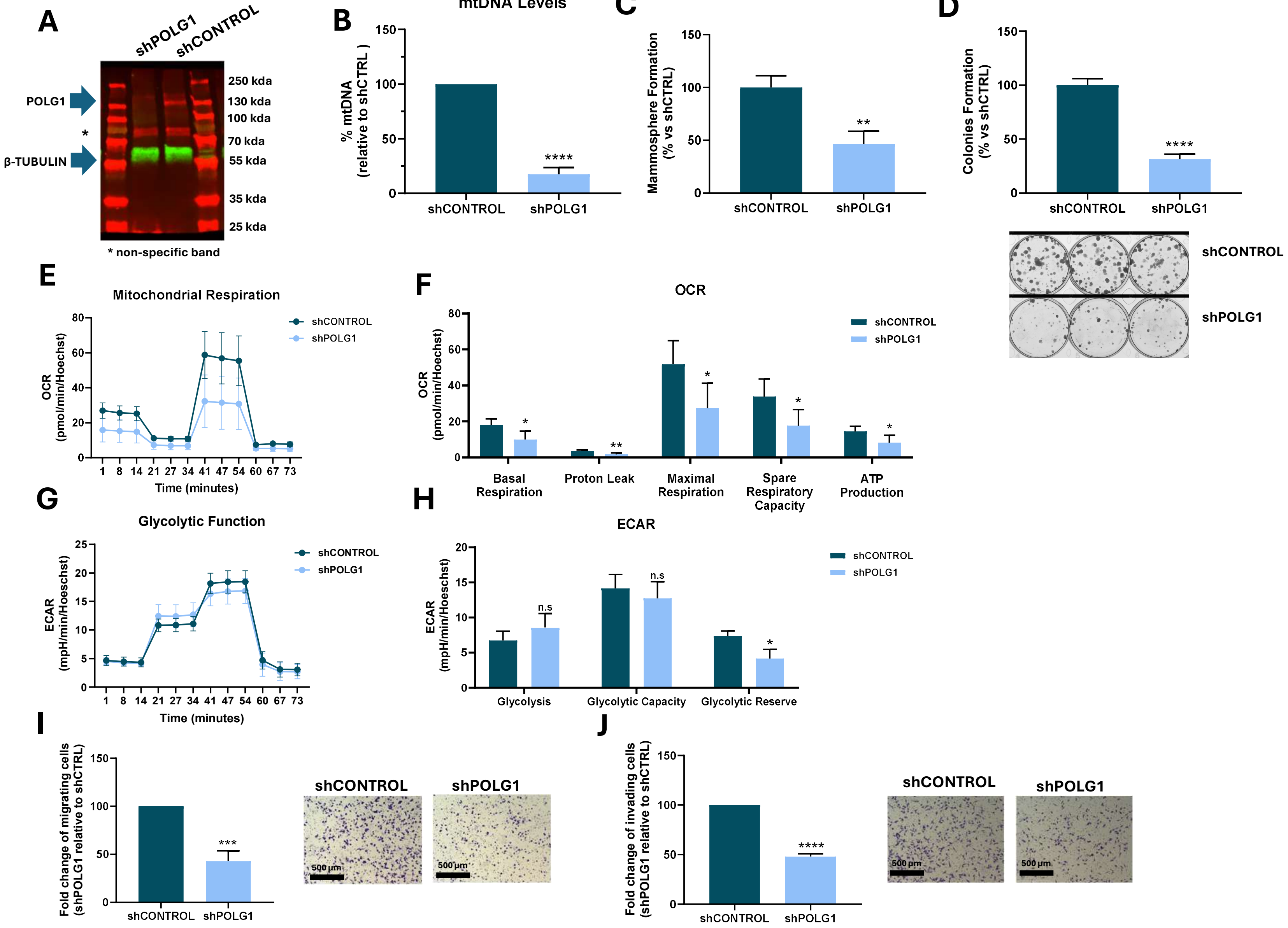

Supplemental Figure S10

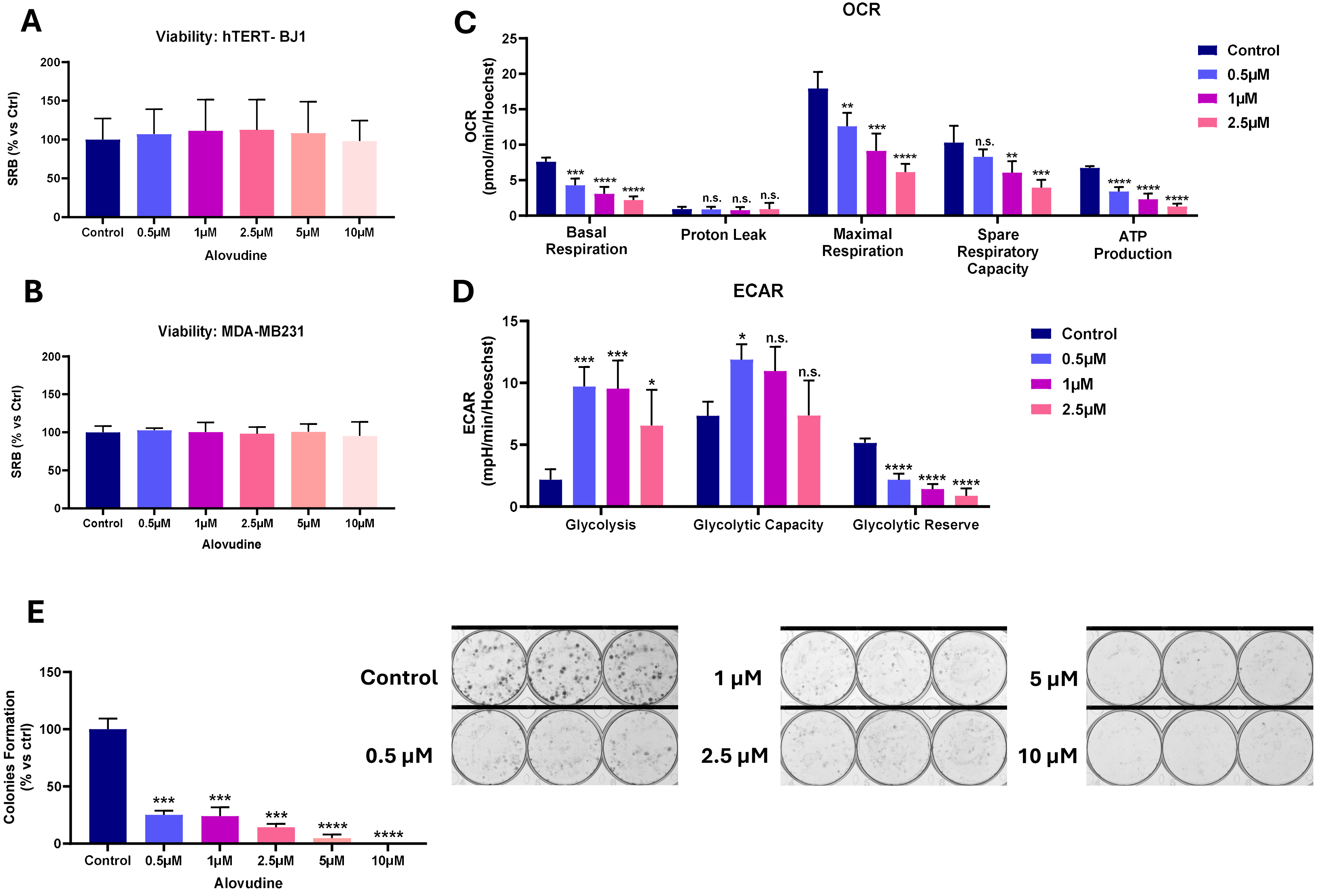

Supplemental Figure S11

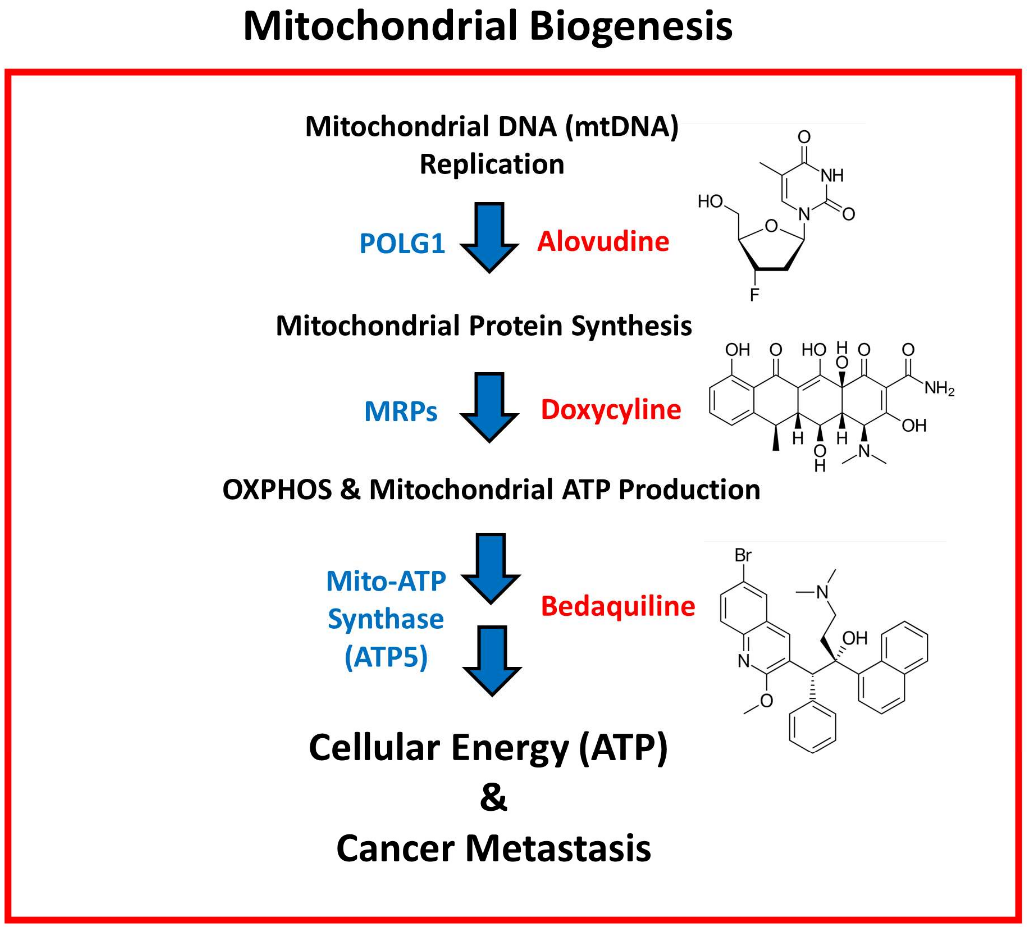

Supplement: Supplementary file 2 — Supplemental Figures [file 41419_2024_7103_MOESM2_ESM.pdf]
