# Supplementary figures and images for: High mitochondrial DNA content is a key determinant of stemness, proliferation, cell migration, and cancer metastasis in vivo
Source: Cell Death Dis. 2024 Oct 11;15(10):745. doi: 10.1038/s41419-024-07103-9 (PMC11470112; doi:10.1038/s41419-024-07103-9)

### Uncropped Western Blots.

Related to Supplemental Figure S9, panel A.

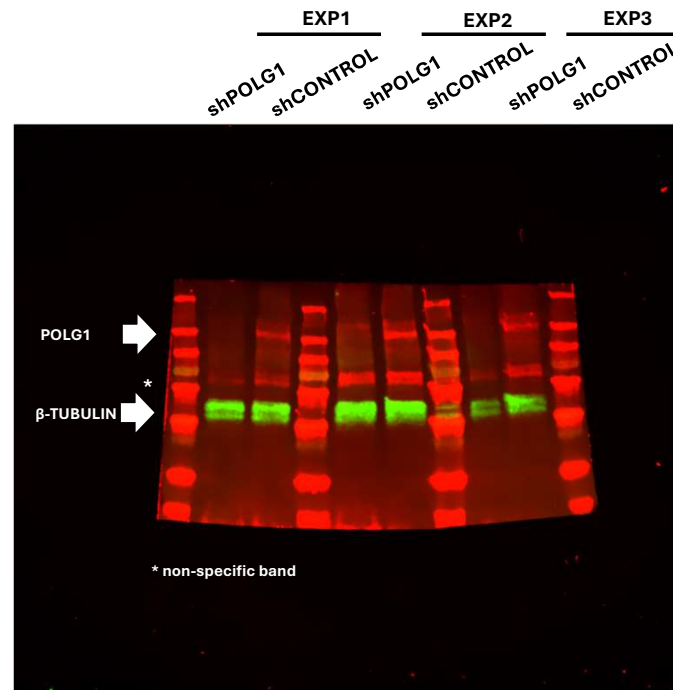

Supplement: Supplementary file 3 — Original Data File [file 41419_2024_7103_MOESM3_ESM.pdf]
